# Association between the Use of Statins and Brain Tumors

**DOI:** 10.3390/biomedicines11082247

**Published:** 2023-08-10

**Authors:** Sarang Jang, Hyo Gun Choi, Mi Jung Kwon, Ji Hee Kim, Joo-Hee Kim, So Young Kim

**Affiliations:** 1 Department of Public Health, Sahmyook University, Seoul 01795, Republic of Korea; jangsarang@gmail.com; 2 Mdanalytics, Seoul 06349, Republic of Korea; pupen@naver.com; 3 Suseoseoulent Clinic, Seoul 06349, Republic of Korea; 4 Department of Pathology, Hallym University Sacred Heart Hospital, Hallym University College of Medicine, Anyang 14068, Republic of Korea; mulank99@hallym.or.kr; 5 Department of Neurosurgery, Hallym University Sacred Heart Hospital, Hallym University College of Medicine, Anyang 14068, Republic of Korea; kimjihee@hallym.or.kr; 6 Department of Medicine, Hallym University Sacred Heart Hospital, Hallym University College of Medicine, Anyang 14068, Republic of Korea; luxjhee@hallym.or.kr; 7 Department of Otorhinolaryngology-Head & Neck Surgery, CHA Bundang Medical Center, CHA University, Seongnam 13496, Republic of Korea

**Keywords:** brain neoplasms, Hydroxymethylglutaryl-CoA reductase inhibitors, cohort studies, case–control studies, epidemiology

## Abstract

This study aimed to investigate the effects of statin use on the incidence of brain tumors. The Korean National Health Insurance Service—National Sample Cohort from 2005 to 2019 was used. The 1893 patients who were diagnosed with brain tumors were matched with 7572 control patients for demographic variables. The history of dyslipidemia was collected, and their history of prescription of statins before diagnosis of brain tumor was examined. The participants without dyslipidemia were set as a reference population. Then, the odds for brain tumors were analyzed in dyslipidemia patients without statin use, dyslipidemia patients who were prescribed statins for less than 365 days, and dyslipidemia patients who were prescribed statins for 365 days or more. Propensity score overlap weighted multivariable logistic regression analysis was used and adjusted for demographics and comorbidities. Secondary analyses were conducted according to types of statins, malignancy of brain tumors, and histories of demographics or comorbidities. A total of 11.78% of brain tumor patients and 10.95% of control participants had histories of statin use for 365 days or more. Dyslipidemia patients with 365 days or more duration of statin use demonstrated 1.22 times higher odds for brain tumors than normal participants (95% confidence intervals [CI] = 1.06–1.14, *p* = 0.007). Dyslipidemia patients with less than 365 days of statin use had higher odds of brain tumors than other groups (odds ratio = 1.60, 95% CI = 1.36–1.87, *p* < 0.001). The higher odds for brain tumors in short-term statin users (<365 days) than in long-term statin users (≥365 days) were consistent in secondary analyses according to types of statins, malignancy of brain tumors, and histories of demographics or comorbidities. Long-term statin use in dyslipidemia patients was related to a lower risk of brain tumors than short-term statin use in patients with dyslipidemia.

## 1. Introduction

Statins are widely prescribed drugs for lowering cholesterol. They repress the function of HMG-CoA reductase, which is known as a rate-limiting step for synthesizing cholesterol. In addition to regulating cholesterol levels, statins have a role in the cardiovascular system via anti-inflammatory effects and immune modulation [1,2,3]. Statins inhibit mevalonate pathway, in that reducing the prenylation of Rho proteins which activate nitric oxide synthase expression [3]. In addition, statins inhibit the expression of Major histocompatibility complex class II molecules, which mediate the T-cell activation [3]. The regulatory effects on inflammation and immune responses may have effects on other diseases. Indeed, in addition to the cardiovascular system, statins have been suggested to have beneficial effects on a variety of diseases, including endocrine diseases such as thyroid disease and polycystic ovarian syndrome and neurological disorders such as Alzheimer’s disease, Parkinson’s disease, multiple sclerosis, and primary brain tumors [4,5]. A number of previous studies have suggested the anticancer effects of statins [6,7]. In particular, a number of previous studies have suggested the anticancer effects of statins in brain tumor patients [8]. However, clinical studies on the anticancer effects of statins on cancer are still controversial. Moreover, the detrimental effects of statins on cancer cells have been suggested to elevate the risk of cancer cell seeding by inducing epithelial-to-mesenchymal transition and heterogeneous responses [9]. This discrepancy may originate from different pathophysiologic mechanisms according to the specific types of cancer. For instance, brain tumors mainly are not of epithelial origin. Thus, the impact of statins may be different from other types of tumors.

Brain tumors are one of the most fatal malignancies and cause considerable morbidity and mortality. According to a survey from the Central Brain Tumor Registry of the United States, the incidence of malignant brain tumors was estimated to be approximately 0.8% per year in all age populations, and the 5-year survival rate was calculated to be approximately 36% [10]. Research on the risk factors for brain tumors has been limited due to the rarity and heterogeneity of brain tumors. A history of ionizing radiation is a well-known risk factor for primary brain tumors, which were mainly meningiomas and gliomas in both adults and children [11]. In these histologic types of brain tumors, nutritional and environmental factors, such as nitrosamines, protein intake, and hyperlipidemia, have been proposed to increase the risk of brain tumors [12,13]. Nitrosamine was supposed to interact with the protein intake, which was suggested to increase the risk of meningiomas and gliomas [12]. In patients with high-grade glioma, high level of serum cholesterol and increase of LDL level were associated with poor survival outcomes [13]. The most common types of brain tumors are known as intracranial metastases from other primary malignancies, followed by meningiomas and gliomas [14]. Glioblastoma is the most prevalent malignant primary brain tumor and requires concurrent chemoradiation due to its aggressive progression. Several previous studies have suggested the therapeutic effects of statins in brain tumors, such as glioblastoma [15]. However, there has been no consensus on the clinical evidence for the effects of statins on brain tumors.

We hypothesized that statins could attenuate the risk of brain tumors in patients with dyslipidemia. To test this hypothesis, we compared the rates of brain tumors according to the history of statin use in dyslipidemia patients. The duration of statin use was divided into short-term (<365 days) and long-term (≥365 days) user groups, and the rates of brain tumors were estimated in each group. Although the pathophysiologic mechanisms need to be elucidated, dyslipidemia was suggested to be related to the incidence of brain tumors [13]. One of the reasons for this may be the abnormal lipid metabolism and high level of cholesterol in glioma patients [13]. Thus, the presence of dyslipidemia was included and analyzed for its association with the incidence of brain tumors.

## 2. Methods

### 2.1. Exposure (Dyslipidemia and Statin)

Dyslipidemia (ICD-10 codes: E78) was defined for the participants who visited clinics with diagnosis of dyslipidemia ≥ 2 times. Statin use was defined as prescription within 2 years before the index dates in both brain tumor and control participants. The participants were grouped into four groups: (1) normal (which is reference), (2) dyslipidemia without statin, (3) dyslipidemia with statin prescription dates < 365 days, and (4) dyslipidemia with 365 days ≤ statin prescription dates.

### 2.2. Outcome (Brain Tumors)

Brain tumors were defined as malignant brain tumors (Korean Standard Classification of Diseases [KCD] codes: C71, C75.1) and benign brain tumors (KCD codes: D33.0, D33.1, D33.2, D33.3, D35.2, and D35.3). Among them, we selected participants who had a special claim code for cancer (V193 and V194).

### 2.3. Participant Selection

The Korean National Health Insurance Service—National Sample Cohort (NHIS-NSC, from 2002 to 2019) was used for this study [16,17].

**Figure 1:**
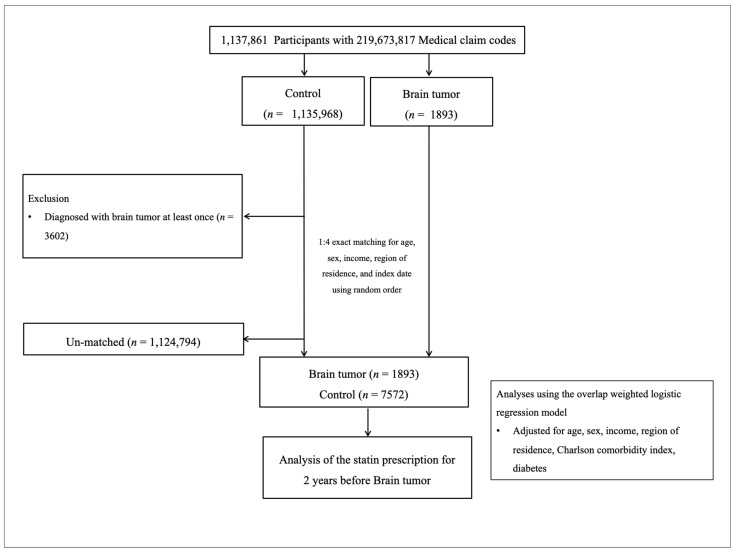
A schematic illustration of the participant selection process used in the present study. Of a total of 1,137,861 participants, 1893 brain tumor participants were matched with 7572 control participants for age, sex, income, and region of residence.

Brain tumor participants were selected from 1,137,861 participants with 219,673,817 medical claim codes from 2005 through 2019 (*n* = 1893). The control group was included if participants were not defined as having brain tumors from 2005 through 2019 (*n* = 1,135,968). The participants who were diagnosed with brain tumors at least once (*n* = 3602) were excluded for controls. Brain tumor participants and control participants were equalized for age, sex, income, and region of residence. The initial diagnosed date of each brain tumor participant was set as the index date. The matched control participants were set as the identical index date of the paired brain tumor participants. The 1,124,794 control participants were excluded due to no matched brain tumor participants. Finally, 1893 brain tumor participants and 7572 control participants were included in the analyses (Figure 1).

### 2.4. Covariates

Age groups were classified as 0–4, 5–9, 10–14, …, and 85+ years old. Income groups were divided into classes 1 (lowest income) to 5 (highest income). The urban and rural areas were grouped [18,19].

The Charlson Comorbidity Index (CCI) measures disease burden based on the medical histories of 17 comorbidities. Depending on the number of comorbidities, CCI was estimated as the continuous variable [20,21]. Diabetes was excluded from the calculation of CCI score.

Diabetes (claim codes: E10–E14) was separately assessed based on ≥2 clinical visits.

### 2.5. Statistical Analyses

Propensity score overlap weighting was conducted to balance the covariate and effective sample size. The propensity score (PS) was estimated by multivariable logistic regression. To compute overlap weighting, case participants were weighted by the probability of 1–PS and control participants were weighted by the probability of PS. Overlap weighting was applied between 0 and 1 [22,23,24]. The general characteristics were compared between the brain tumor and control groups using standardized difference (sd).

Propensity score overlap weighted multivariable logistic regression analysis was used and calculated the overlap weighted odds ratios (ORs) of statin prescriptions for any brain tumor, malignant brain tumors, and benign brain tumors. In these analyses, crude and overlap weighted models were applied. Additionally, statin prescriptions were analyzed by dividing the patients into any statin, lipophilic statin, and hydrophilic statin groups. In addition, subgroup analyses according to age, sex, income, region of residence, CCI, and diabetes were performed.

The 95% confidence interval (CI) was calculated. Two-tailed analyses were performed. *p* values less than 0.05 were set as statistical significance. SAS version 9.4 (SAS Institute Inc., Cary, NC, USA) was used for statistical analyses.

## 3. Results

The rates of statin use were higher in patients with brain tumors than in control participants (Table 1). A total of 13.42% (254/1893) and 11.78% (223/1893) of patients with brain tumors and 7.82% (592/7572) and 10.95% (829/7572) of control participants had histories of <365 days and ≥365 days of statin use, respectively (sd = 0.19). When classifying the history of statin use according to the presence of dyslipidemia, 18.44% (349/1893), 9.72% (184/1893), and 10.57% (200/1893) of brain tumor patients with dyslipidemia had none, <365 days, and ≥365 days of statin use, respectively. In control participants, 24.56% (1860/7572), 6.72% (509/7572), and 9.85% (746/7572) of brain tumor patients with dyslipidemia had none, <365 days, and ≥365 days of statin use, respectively. The CCI score and diabetes history were different between the brain tumor and control groups. After PS overlap weighting adjustment, there was no difference in these variables between the two groups (sd = 0.00).

Compared to the participants without dyslipidemia, the dyslipidemia patients without statins demonstrated low odds for brain tumors (OR = 0.81, 95% CI = 0.73–0.90, *p* < 0.001, Table 2). According to the types of statins, dyslipidemia patients without lipophilic statins showed low odds for brain tumors (OR = 0.86, 95% CI = 0.77–0.95, *p* = 0.002). On the other hand, dyslipidemia patients with <365 days of statin use had ORs as high as 1.60 for brain tumors (95% CI = 1.36–1.87, *p* < 0.001). A longer duration (≥365 days) of statin use in dyslipidemia patients was related to lower odds for brain tumors than a short duration of statin use (OR = 1.22, 95% CI = 1.06–1.14, *p* = 0.007). Both lipophilic and hydrophilic statin use for <365 days were associated with high odds for brain tumors. The long duration of lipophilic or hydrophilic statin use reduced the odds for brain tumors.

Both malignant and benign brain tumors demonstrated similar associations between the incidence of brain tumors and statin use in dyslipidemia. Dyslipidemia patients without statins showed an OR of 0.65 for malignant brain tumors (95% CI = 0.56–0.75, *p* < 0.001, Table 3). The <365 days of statin use in dyslipidemia patients was related to 1.61 times higher odds for malignant brain tumors (95% CI = 1.29–2.00, *p* < 0.001). In contrast, in dyslipidemia patients with ≥365 days of statin use, the odds for malignant brain tumors were not different from those in the participants without dyslipidemia. Both lipophilic and hydrophilic statins showed comparable results.

For benign brain tumors, the odds for benign brain tumors in dyslipidemia patients without statins were not different from those in the participants without dyslipidemia (Table 4). The odds for benign brain tumors were highest in dyslipidemia patients with <365 days of statin use (OR = 1.75, 95% CI = 1.39–2.21, *p* < 0.001).

**Figure 2:**
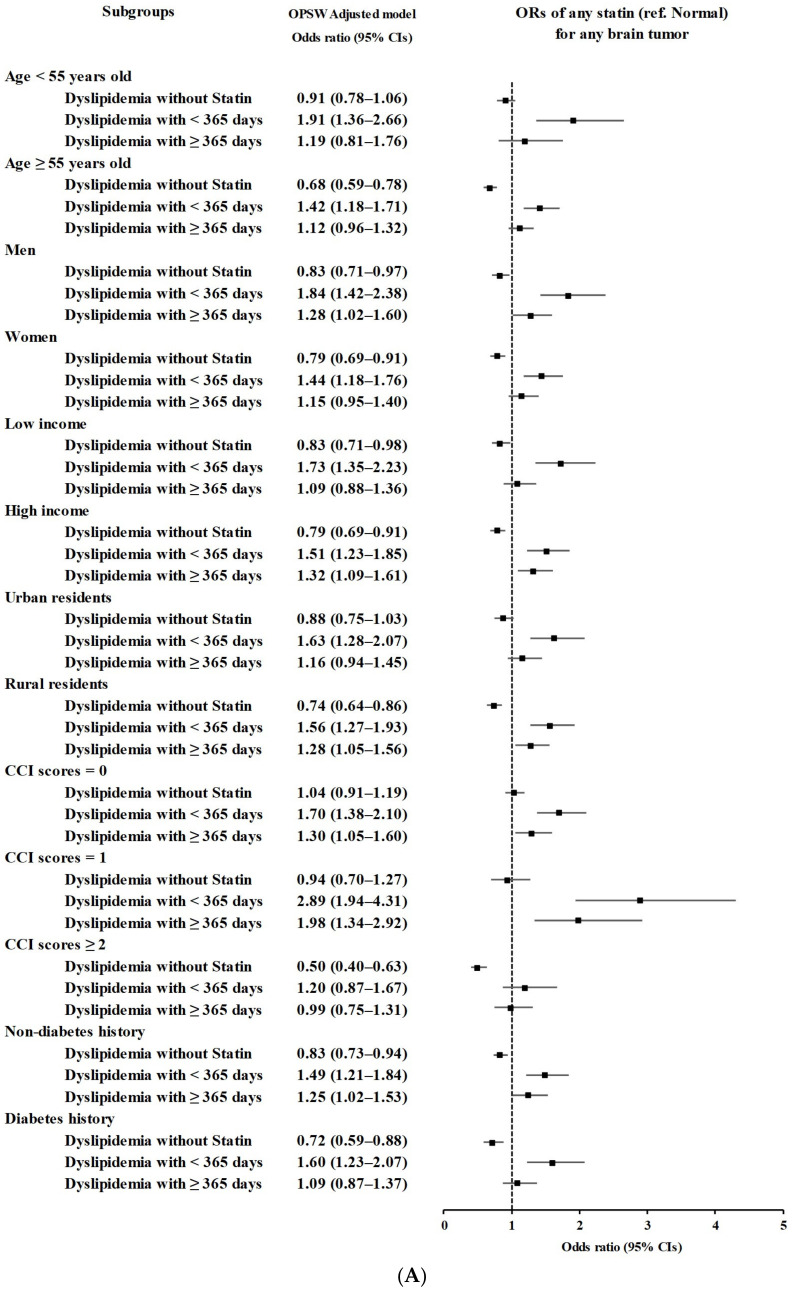

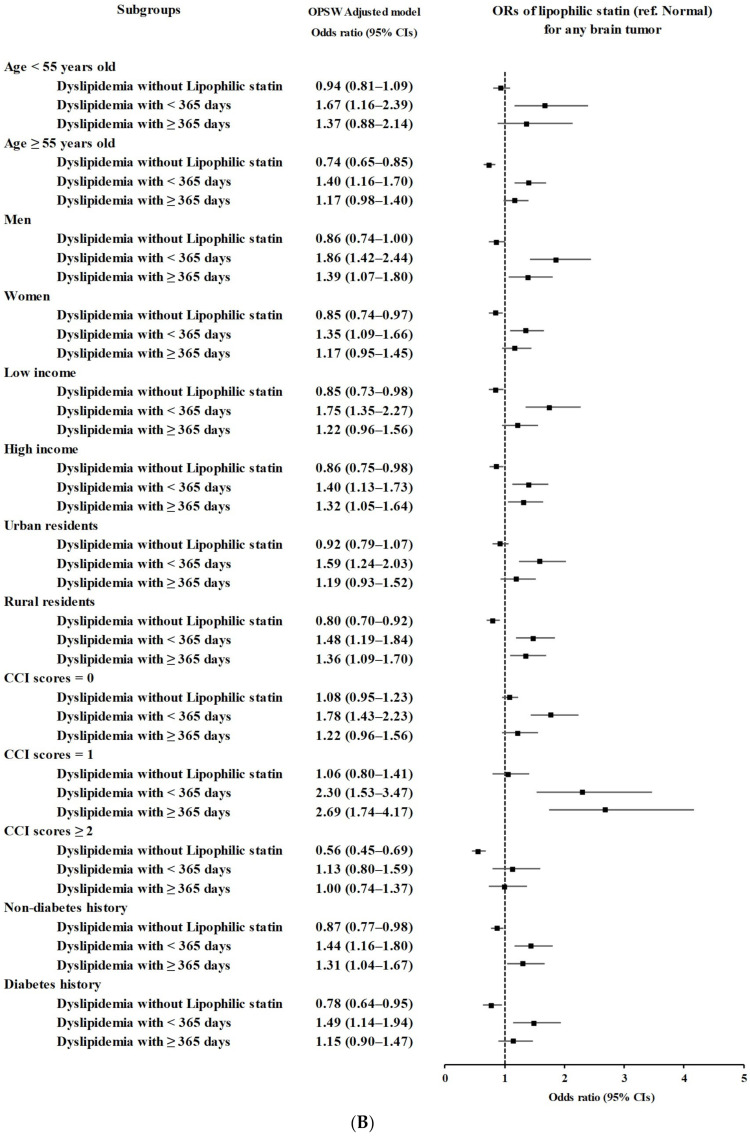

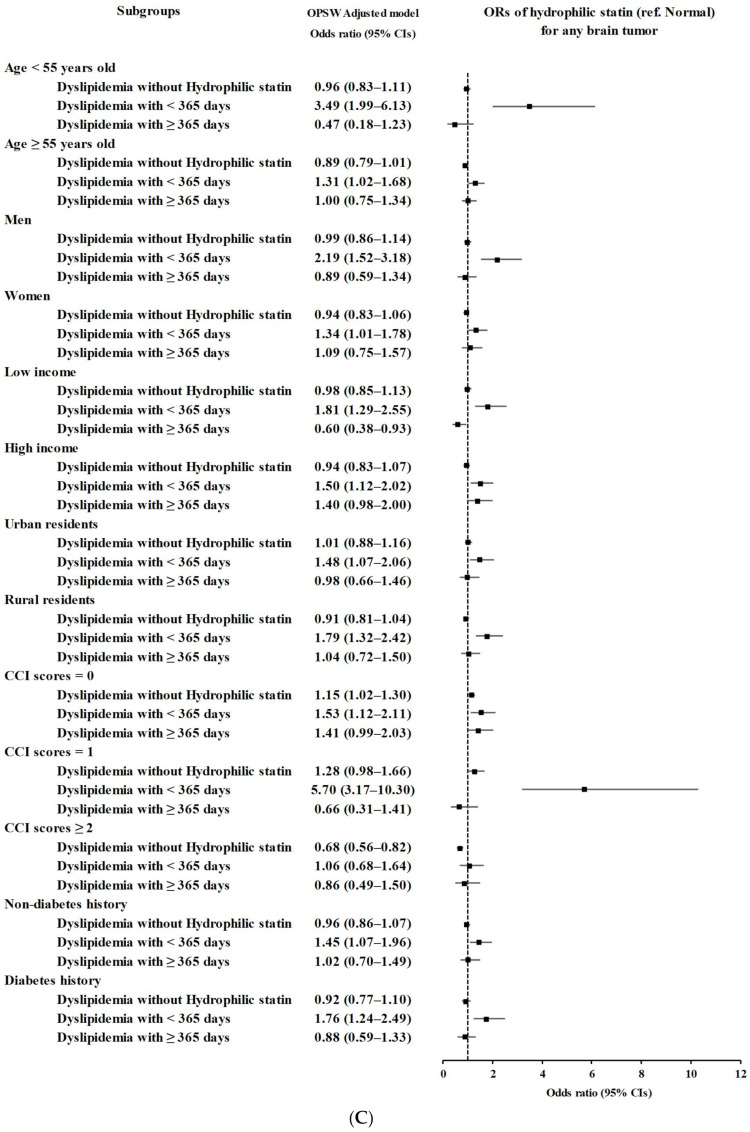

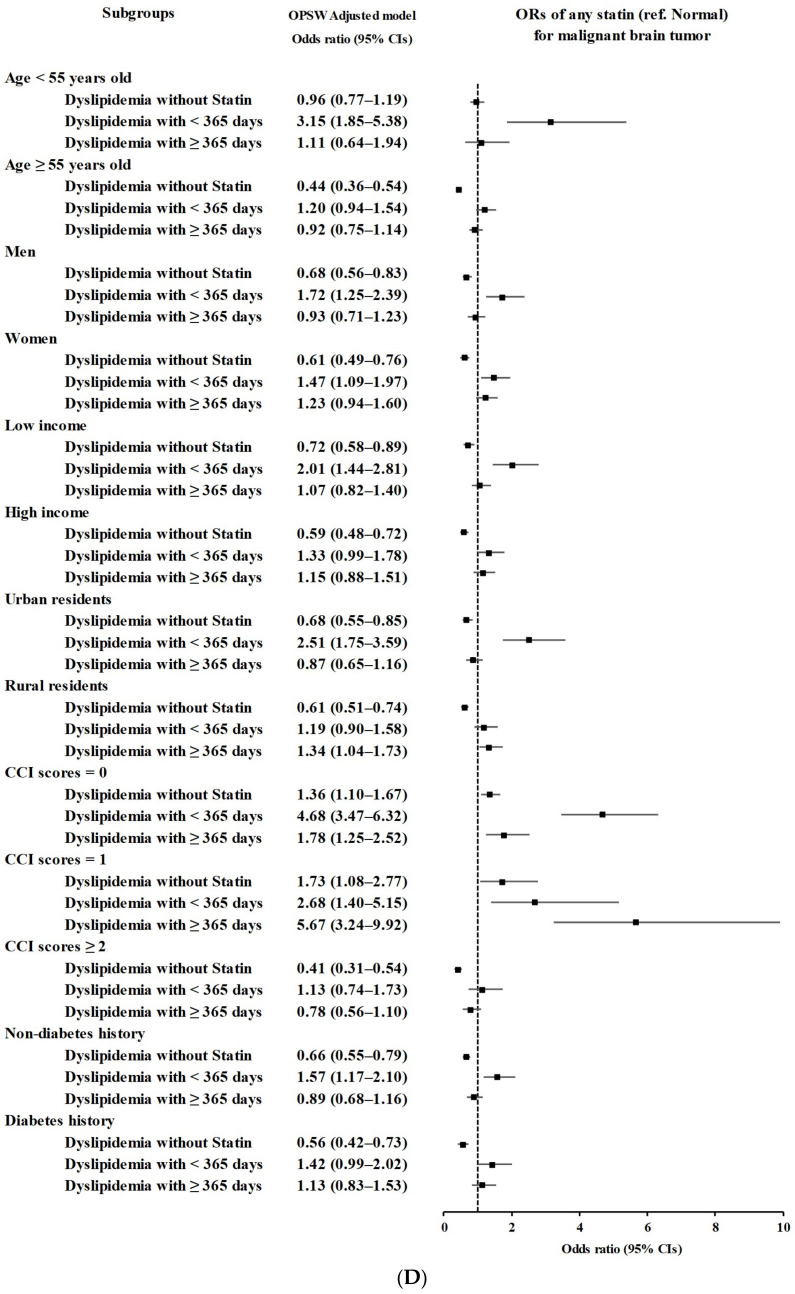

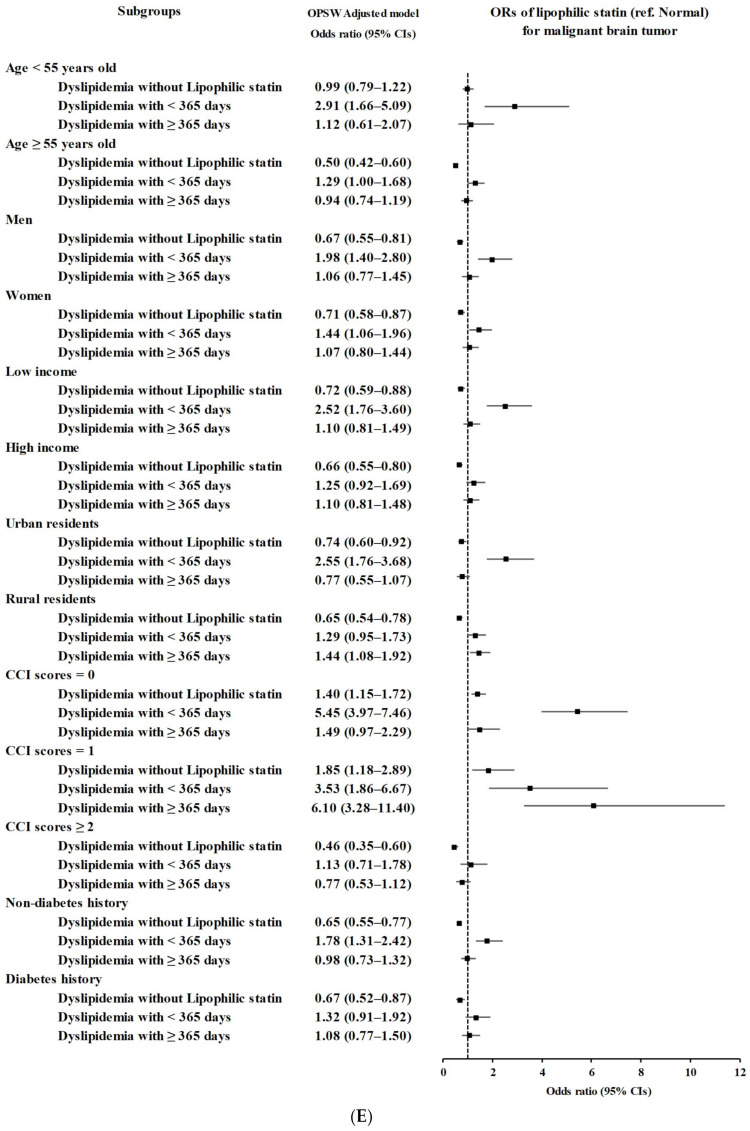

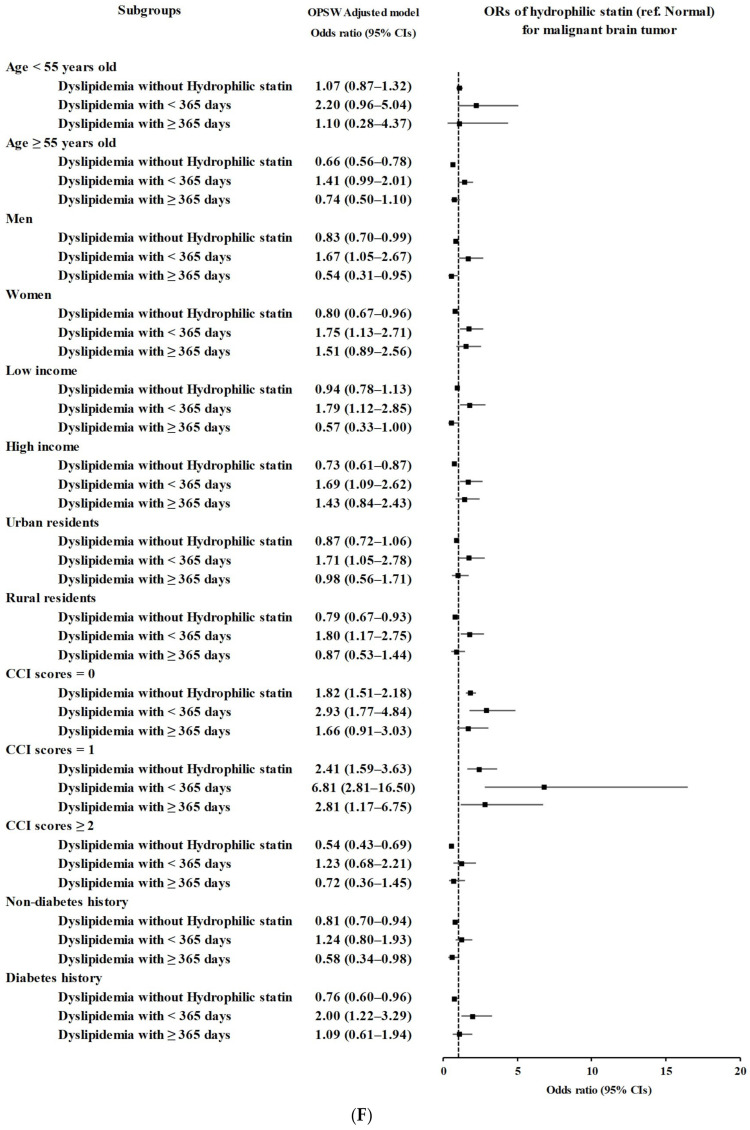

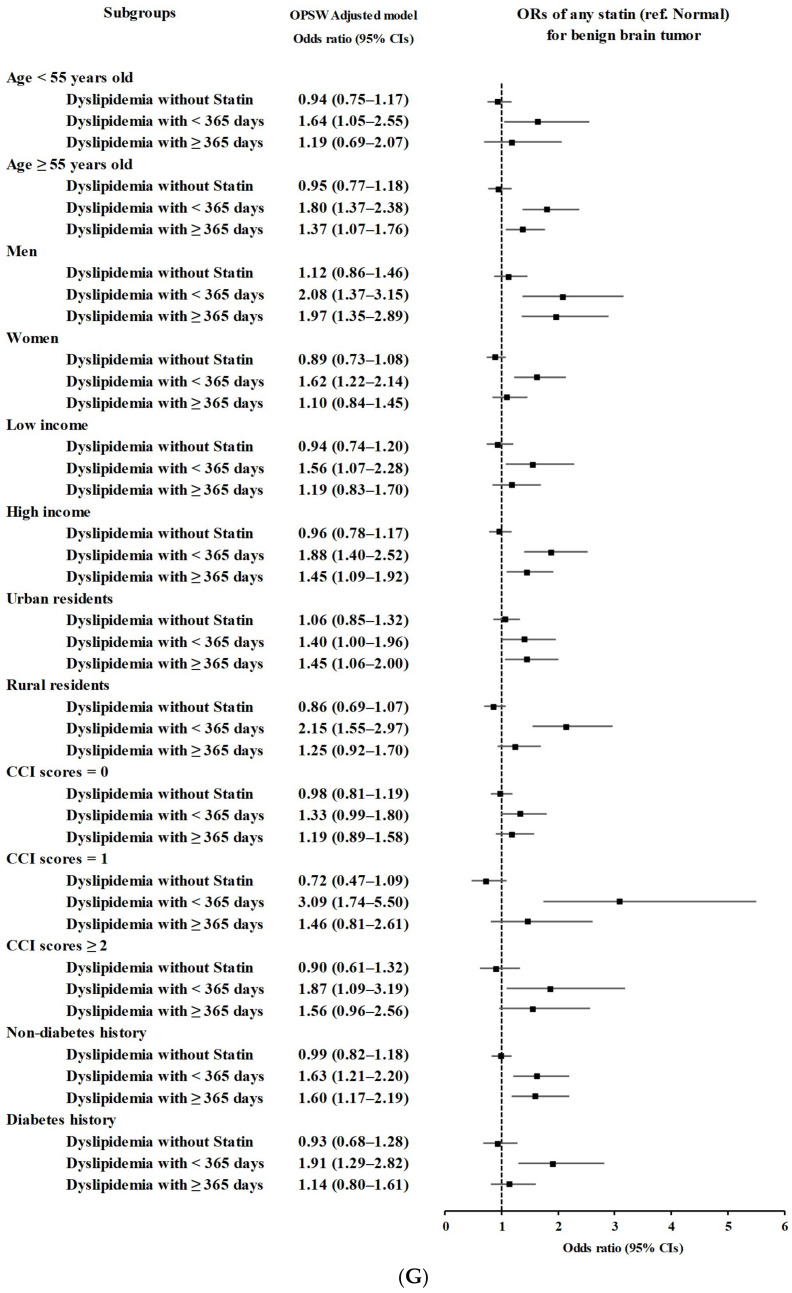

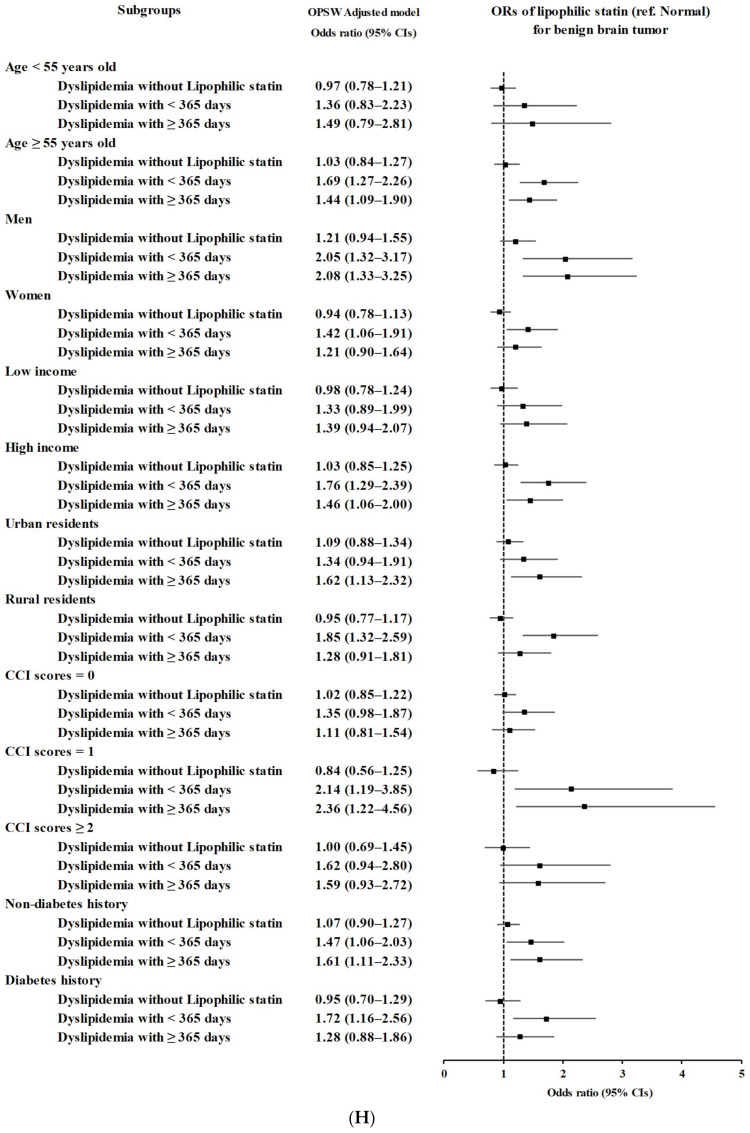

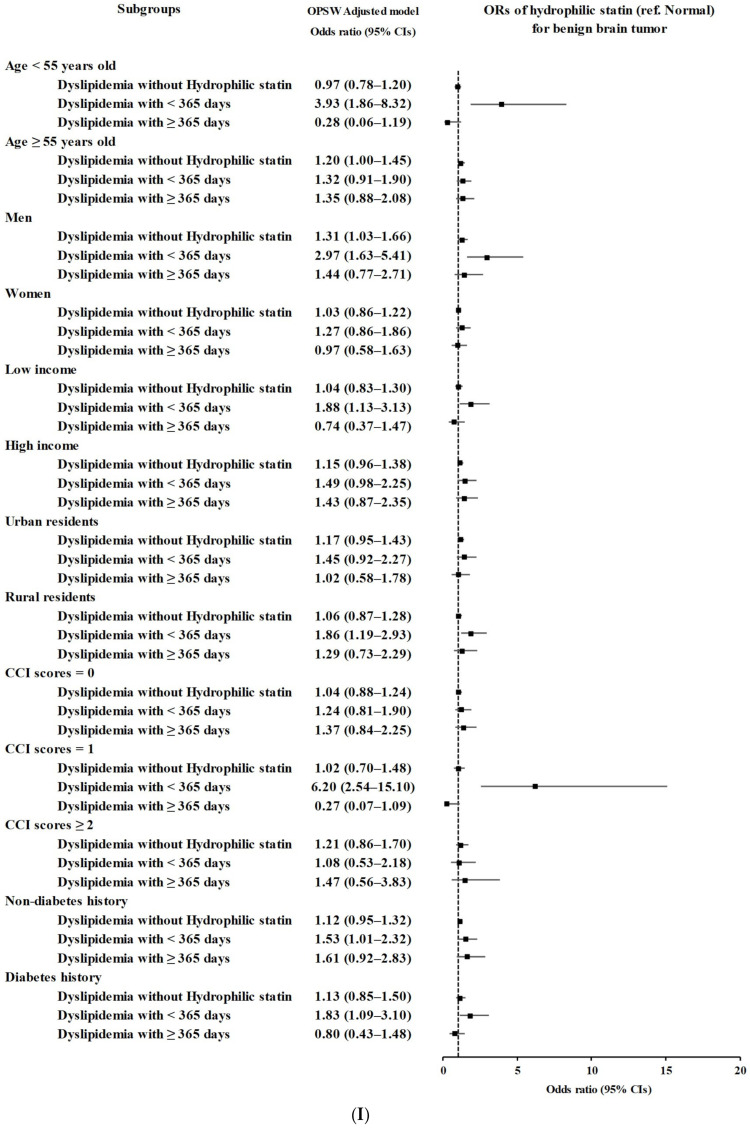
(**A**) Overlap propensity score weighted odds ratios of dates of any statin prescription for any type of brain tumor. (**B**) Overlap propensity score weighted odds ratios of dates of lipophilic statin prescription for any type of brain tumor. (**C**) Overlap propensity score weighted odds ratios of dates of hydrophilic statin prescription for any type of brain tumor. (**D**) Overlap propensity score weighted odds ratios of dates of any type of statin prescription for malignant brain tumors. (**E**) Overlap propensity score weighted odds ratios of dates of lipophilic statin prescription for malignant brain tumors. (**F**) Overlap propensity score weighted odds ratios of dates of hydrophilic statin prescription for malignant brain tumors. (**G**) Overlap propensity score weighted odds ratios of dates of any type of statin prescription for benign brain tumors. (**H**) Overlap propensity score weighted odds ratios of dates of lipophilic statin prescription for benign brain tumors. (**I**) Overlap propensity score weighted odds ratios of dates of hydrophilic statin prescription for benign brain tumors.

The secondary groups according to age, sex, income, region of residence, CCI score, and history of diabetes demonstrated a consistent association of statin use in dyslipidemia patients with the incidence of brain tumors (Figure 2 and Tables S1–S9).

## 4. Discussion

The incidence of brain tumors was high in dyslipidemia patients with a short duration of statin use in the present study. A longer duration of statin use also demonstrated high odds for brain tumors compared to the participants without dyslipidemia. However, the dyslipidemia patients with longer duration of statin use showed lower odds of brain tumors than patients with shorter duration of statin use. The current results implied the protective effects of long-term statin medication for the development of brain tumors. Interestingly, dyslipidemia patients without statin use did not show a high rate of brain tumors in this study. This phenomenon can be partially explained by the fact that these dyslipidemia patients may have mild dyslipidemia and do not need prescriptions for statin medication; thus, their dyslipidemia did not add to the risk of brain tumors. In addition, among normal participants, some may have undiagnosed dyslipidemia, which may increase the risk of brain tumors.

A few prior studies have suggested the protective effects of statins against brain tumors. Statins may exert multiple cascades that suppress brain cancer cells [15]. Preclinical studies have demonstrated that statins seize the cell cycle and result in apoptosis of cancer cells by activation of Bax and deactivation of BCL-2 [25]. In addition, statins deplete isoprenoids, which are required for the prenylation of small Rho GTPases in cancer cells [26]. For instance, lovastatin was reported to inhibit brain tumor stem cells, which may suppress brain tumors [27]. By interfering with the mevalonate pathway, statins were supposed to reduce the proliferation and differentiation of cancer stem cells and accelerate apoptosis [28,29,30]. Moreover, statins suppressed the metastasis of breast cancers [31,32]. Therefore, it can be presumed that statins can improve the prognosis of brain tumors.

However, the clinical effects of statins on brain tumors have been controversial. A number of previous studies have demonstrated an equivocal relationship between statin use and the incidence of brain tumors [33]. A meta-analysis examined 51 preclinical studies that addressed the effects of statins on the proliferation, migration, and invasion of gliomas [34]. However, 13 clinical studies, which were analyzed by meta-analysis, did not show a relationship between statin use and the incidence and survival rate of glioma [34].

The patients with dyslipidemia, except for the patients without statins, in the current study demonstrated higher odds for brain tumors. A few clinical studies have reported that dyslipidemia may increase the risk of brain tumors. In a prospective cohort study using the UK Biobank, total cholesterol levels were related to an increased risk of glioma in men [35]. Moreover, a retrospective study demonstrated that high-grade glioma patients with high serum levels of cholesterol before treatment had a lower 5-year survival rate and median survival time than those with low levels of cholesterol (4.9% and 23.6 months vs. 19.6% and 24.5 months) [13]. A preclinical study reported that cholesterol is crucial for the survival of glioblastoma cells [36]. Indirectly, metabolic complications in dyslipidemia patients may have adverse consequences that make them susceptible to brain tumors. Our results indicated that long-term statin use in these dyslipidemia patients can alleviate vulnerability to brain tumors.

The present study analyzed huge, nationwide population data in Korea. Due to the large size of the study population, we can achieve a sufficient number of control participants with PS overlap weighting adjustment. Because all Koreans are legally registered in the national healthcare system, there was little concern about missing data or selection bias. All diagnostic codes were registered by a physician; thus, the reliability of the medical history was fairly sound. However, because the data stemmed from health claim codes, there may have been some variations on the accuracy of diagnostic coding and some patients could be missed if they did not visit clinics. In addition, the absence of clinical data, including radiologic or pathologic findings of brain tumors, can influence the current findings. Although we classified types of brain tumors as malignant and benign brain tumors, the histologic types and stages of brain tumors were heterogeneous. Brain tumors are a diverse group of diseases with varying etiologies, prognoses, and responses to treatment. Thus, combining them into a single category might dilute the specific associations between statin use and particular types or stages of brain tumors. For statin medication, because this study analyzed the prescription history of statins, the compliance of patients with statin prescriptions can influence the relationship with the incidence of brain tumors. Patients with dyslipidemia may not adhere to a statin prescription due to a number of reasons, such as side effects, cost, or personal beliefs, which can impact the association between statin use and brain tumor in this study. Although this study matched and adjusted both demographic and lifestyle factors and comorbidities to minimize the potential confounding effects, the possibility of confounding effects cannot be completely excluded in the present study. The observational nature of this study could leave room for unmeasured confounders. For example, variables such as a history of ionizing radiation, dietary patterns, physical activity, or genetic predispositions could impact both the use of statins and the risk of brain tumors [13]. Because the study population of this study was Korean, there may be some ethnic differences in the association of statin use with the incidence of brain tumors, in that this can limit the generalizability of the association between statin use and brain tumors in the present study. Finally, because this study had a retrospective study design, the causality between statin use and brain tumors cannot be concluded.

## 5. Conclusions

The presence of dyslipidemia with short-term statin use was related to the 1.6 times higher incidence of brain tumors. However, long-term statin use in dyslipidemia patients was associated with a lower odds of brain tumors (1.22) than short-term statin use in patients with dyslipidemia. This study is a pioneering study that analyzed the association of statin use with the incidence of brain tumors in a large, nationwide cohort population. Moreover, types of statins were considered, and numerous comorbidities were adjusted and grouped to minimize the potential bias from these confounders. The present findings imply the potential role of management of dyslipidemia for development of brain tumors.

## Figures and Tables

**Table 1:** General characteristics of participants.

Characteristics	Before PS Overlap Weighting Adjustment			After PS Overlap Weighting Adjustment		
	Brain Tumor	Control	Standardized Difference	Brain Tumor	Control	Standardized Difference
Age (n, %)			0.00			0.00
0–4	18 (0.95)	72 (0.95)		10 (0.84)	10 (0.84)	
5–9	35 (1.85)	140 (1.85)		21 (1.77)	21 (1.77)	
10–14	39 (2.06)	156 (2.06)		22 (1.87)	22 (1.87)	
15–19	58 (3.06)	232 (3.06)		38 (3.26)	38 (3.26)	
20–24	53 (2.80)	212 (2.80)		34 (2.91)	34 (2.91)	
25–29	41 (2.17)	164 (2.17)		27 (2.33)	27 (2.33)	
30–34	66 (3.49)	264 (3.49)		42 (3.62)	42 (3.62)	
35–39	92 (4.86)	368 (4.86)		56 (4.87)	56 (4.87)	
40–44	147 (7.77)	588 (7.77)		94 (8.11)	94 (8.11)	
45–49	159 (8.40)	636 (8.40)		99 (8.59)	99 (8.59)	
50–54	189 (9.98)	756 (9.98)		118 (10.24)	118 (10.24)	
55–59	178 (9.40)	712 (9.40)		110 (9.54)	110 (9.54)	
60–64	186 (9.83)	744 (9.83)		116 (10.02)	116 (10.02)	
65–69	213 (11.25)	852 (11.25)		125 (10.85)	125 (10.85)	
70–74	177 (9.35)	708 (9.35)		104 (9.03)	104 (9.03)	
75–79	134 (7.08)	536 (7.08)		79 (6.84)	79 (6.84)	
80–84	78 (4.12)	312 (4.12)		44 (3.82)	44 (3.82)	
85+	30 (1.58)	120 (1.58)		17 (1.50)	17 (1.50)	
Sex (n, %)			0.00			0.00
Male	855 (45.17)	3420 (45.17)		509 (44.01)	509 (44.01)	
Female	1038 (54.83)	4152 (54.83)		647 (55.99)	647 (55.99)	
Income (n, %)			0.00			0.00
1 (lowest)	344 (18.17)	1376 (18.17)		204 (17.63)	204 (17.63)	
2	218 (11.52)	872 (11.52)		132 (11.42)	132 (11.42)	
3	290 (15.32)	1160 (15.32)		177 (15.30)	177 (15.30)	
4	449 (23.72)	1796 (23.72)		275 (23.80)	275 (23.80)	
5 (highest)	592 (31.27)	2368 (31.27)		368 (31.85)	368 (31.85)	
Region of residence (n, %)			0.00			0.00
Urban	849 (44.85)	3396 (44.85)		525 (45.41)	525 (45.41)	
Rural	1044 (55.15)	4176 (55.15)		631 (54.59)	631 (54.59)	
CCI score (Mean, SD)	2.57 (2.44)	0.54 (1.21)	1.05	1.47 (1.31)	1.47 (0.84)	0.00
Diabetes history (n, %)	526 (27.79)	1898 (25.07)	0.06	311 (26.90)	311 (26.90)	0.00
Dyslipidemia and Statin prescription dates (n, %)			0.17			0.18
Normal	1160 (61.28)	4457 (58.86)		677 (58.51)	678 (58.64)	
Dyslipidemia without Statin	349 (18.44)	1860 (24.56)		229 (19.84)	290 (25.08)	
Dyslipidemia with <365 days	184 (9.72)	509 (6.72)		119 (10.31)	77 (6.65)	
Dyslipidemia with ≥365 days	200 (10.57)	746 (9.85)		131 (11.35)	111 (9.63)	
Dyslipidemia and Lipophilic Statin prescription dates (n, %)			0.16			0.15
Normal	1160 (61.28)	4457 (58.86)		677 (58.51)	678 (58.64)	
Dyslipidemia without Lipophilic Statin	418 (22.08)	2131 (28.14)		276 (23.83)	328 (28.36)	
Dyslipidemia with <365 days	163 (8.61)	461 (6.09)		105 (9.05)	70 (6.05)	
Dyslipidemia with ≥365 days	152 (8.03)	523 (6.91)		100 (8.62)	81 (6.96)	
Dyslipidemia and Hydrophilic Statin prescription dates (n, %)			0.11			0.10
Normal	1160 (61.28)	4457 (58.86)		677 (58.51)	678 (58.64)	
Dyslipidemia without Hydrophilic Statin	607 (32.07)	2698 (35.63)		397 (34.36)	417 (36.05)	
Dyslipidemia with <365 days	85 (4.49)	222 (2.93)		55 (4.75)	34 (2.92)	
Dyslipidemia with ≥365 days	41 (2.17)	195 (2.58)		28 (2.38)	28 (2.39)	
Dyslipidemia (n, %)	733 (38.72)	3115 (41.14)	0.05	480 (41.49)	478 (41.36)	0.00
Statin prescription dates (n, %)			0.19			0.18
Non-user	1416 (74.80)	6151 (81.23)		860 (74.35)	936 (80.94)	
<365 days	254 (13.42)	592 (7.82)		153 (13.19)	94 (8.12)	
≥365 days	223 (11.78)	829 (10.95)		144 (12.46)	127 (10.94)	
Lipophilic Statin prescription dates (n, %)			0.18			0.16
Non-user	1498 (79.13)	6449 (85.17)		914 (79.02)	979 (84.63)	
<365 days	224 (11.83)	531 (7.01)		132 (11.41)	85 (7.39)	
≥365 days	171 (9.03)	592 (7.82)		111 (9.57)	92 (7.99)	
Hydrophilic Statin prescription dates (n, %)			0.10			0.10
Non-user	1747 (92.29)	7114 (93.95)		1063 (91.93)	1086 (93.92)	
<365 days	101 (5.34)	250 (3.30)		64 (5.52)	40 (3.42)	
≥365 days	45 (2.38)	208 (2.75)		29 (2.54)	31 (2.66)	

Abbreviations: CCI, Charlson Comorbidity Index; PS, Propensity score; N/A, Not applicable.

**Table 2:** Crude and overlap propensity score weighted odds ratios of dates of statin prescription for any brain tumor.

Characteristics	N of Brain Tumors	N of Controls	Odds Ratios for Any Brain Tumor (95% Confidence Interval)			
	(Exposure/Total, %)	(Exposure/Total, %)	Crude	*p*-Value	Overlap Weighted Model †	*p*-Value
Dyslipidemia and Any statin prescription						
Normal	1160/1893 (61.28)	4457/7572 (58.86)	1		1	
Dyslipidemia without Statin	349/1893 (18.44)	1860/7572 (24.56)	0.72 (0.63–0.82)	<0.001 *	0.81 (0.73–0.90)	<0.001 *
Dyslipidemia with <365 days	184/1893 (9.72)	509/7572 (6.72)	1.39 (1.16–1.66)	<0.001 *	1.60 (1.36–1.87)	<0.001 *
Dyslipidemia with ≥365 days	200/1893 (10.57)	746/7572 (9.85)	1.03 (0.87–1.22)	0.731	1.22 (1.06–1.41)	0.007 *
Dyslipidemia and Lipophilic statin prescription						
Normal	1160/1893 (61.28)	4457/7572 (58.86)	1		1	
Dyslipidemia without Lipophilic statin	418/1893 (22.08)	2131/7572 (28.14)	0.75 (0.67–0.85)	<0.001 *	0.86 (0.77–0.95)	0.002 *
Dyslipidemia with <365 days	163/1893 (8.61)	461/7572 (6.09)	1.36 (1.12–1.64)	0.002 *	1.53 (1.30–1.81)	<0.001 *
Dyslipidemia with ≥365 days	152/1893 (8.03)	523/7572 (6.91)	1.12 (0.92–1.35)	0.259	1.28 (1.08–1.51)	0.003 *
Dyslipidemia and Hydrophilic statin prescription						
Normal	1160/1893 (61.28)	4457/7572 (58.86)	1		1	
Dyslipidemia without Hydrophilic statin	607/1893 (32.07)	2698/7572 (35.63)	0.86 (0.78–0.96)	0.009 *	0.96 (0.87–1.05)	0.384
Dyslipidemia with <365 days	85/1893 (4.49)	222/7572 (2.93)	1.47 (1.14–1.91)	0.003 *	1.64 (1.31–2.05)	<0.001 *
Dyslipidemia with ≥365 days	41/1893 (2.17)	195/7572 (2.58)	0.81 (0.57–1.14)	0.223	1.00 (0.77–1.32)	0.972

Abbreviations: CCI, Charlson Comorbidity Index; * Significance at *p* < 0.05. † Adjusted for age, sex, income, region of residence, diabetes history, CCI scores, and diabetes.

**Table 3:** Crude and overlap propensity score weighted odds ratios of dates of statin prescription for malignant brain tumors.

Characteristics	N of Brain Tumors	N of Controls	Odds Ratios for Malignant Brain Tumors (95% Confidence Interval)			
	(Exposure/Total, %)	(Exposure/Total, %)	Crude	*p*-Value	Overlap Weighted Model †	*p*-Value
Dyslipidemia and Any statin prescription						
Normal	725/1072 (67.63)	2596/4288 (60.54)	1		1	
Dyslipidemia without statin	158/1072 (14.74)	995/4288 (23.2)	0.57 (0.47–0.69)	<0.001 *	0.65 (0.56–0.75)	<0.001 *
Dyslipidemia with <365 days	88/1072 (8.21)	278/4288 (6.48)	1.13 (0.88–1.46)	0.333	1.61 (1.29–2.00)	<0.001 *
Dyslipidemia with ≥365 days	101/1072 (9.42)	419/4288 (9.77)	0.86 (0.68–1.09)	0.214	1.10 (0.91–1.34)	0.309
Dyslipidemia and Lipophilic statin prescription						
Normal	725/1072 (67.63)	2596/4288 (60.54)	1		1	
Dyslipidemia without Lipophilic statin	189/1072 (17.63)	1150/4288 (26.82)	0.59 (0.49–0.70)	<0.001 *	0.69 (0.60–0.79)	<0.001 *
Dyslipidemia with <365 days	83/1072 (7.74)	253/4288 (5.9)	1.17 (0.90–1.53)	0.227	1.71 (1.36–2.15)	<0.001 *
Dyslipidemia with ≥365 days	75/1072 (7.00)	289/4288 (6.74)	0.93 (0.71–1.21)	0.59	1.09 (0.88–1.35)	0.407
Dyslipidemia and Hydrophilic statin prescription						
Normal	725/1072 (67.63)	2596/4288 (60.54)	1		1	
Dyslipidemia without Hydrophilic statin	285/1072 (26.59)	1467/4288 (34.21)	0.70 (0.60–0.81)	<0.001 *	0.82 (0.73–0.93)	0.002 *
Dyslipidemia with <365 days	42/1072 (3.92)	113/4288 (2.64)	1.33 (0.93–1.92)	0.123	1.75 (1.27–2.41)	<0.001 *
Dyslipidemia with ≥365 days	20/1072 (1.87)	112/4288 (2.61)	0.64 (0.39–1.04)	0.07	0.92 (0.64–1.34)	0.676

Abbreviations: CCI, Charlson Comorbidity Index; * Significance at *p* < 0.05; † Adjusted for age, sex, income, region of residence, diabetes history, CCI scores, and diabetes.

**Table 4:** Crude and overlap propensity score weighted odds ratios of dates of statin prescription for benign brain tumors.

Characteristics	N of Brain Tumors	N of Controls	Odds Ratios for Benign Brain Tumors (95% Confidence Interval)			
	(Exposure/Total, %)	(Exposure/Total, %)	Crude	*p*-Value	Overlap Weighted Model †	*p*-Value
Dyslipidemia and Any statin prescription						
Normal	435/821 (52.98)	1861/3284 (56.67)	1		1	
Dyslipidemia without statin	191/821 (23.26)	865/3284 (26.34)	0.94 (0.78–1.14)	0.553	0.95 (0.82–1.11)	0.556
Dyslipidemia with <365 days	96/821 (11.69)	231/3284 (7.03)	1.78 (1.37–2.31)	<0.001 *	1.75 (1.39–2.21)	<0.001 *
Dyslipidemia with ≥365 days	99/821 (12.06)	327/3284 (9.96)	1.30 (1.01–1.66)	0.041 *	1.34 (1.08–1.68)	0.009 *
Dyslipidemia and Lipophilic statin prescription						
Normal	435/821 (52.98)	1861/3284 (56.67)	1		1	
Dyslipidemia without Lipophilic statin	229/821 (27.89)	981/3284 (29.87)	1.00 (0.84–1.19)	0.988	1.02 (0.88–1.18)	0.84
Dyslipidemia with <365 days	80/821 (9.74)	208/3284 (6.33)	1.65 (1.25–2.17)	<0.001 *	1.59 (1.24–2.03)	<0.001 *
Dyslipidemia with ≥365 days	77/821 (9.38)	234/3284 (7.13)	1.41 (1.07–1.86)	0.016 *	1.44 (1.12–1.84)	0.004 *
Dyslipidemia and Hydrophilic statin prescription						
Normal	435/821 (52.98)	1861/3284 (56.67)	1		1	
Dyslipidemia without Hydrophilic statin	322/821 (39.22)	1231/3284 (37.48)	1.12 (0.95–1.31)	0.171	1.11 (0.96–1.27)	0.151
Dyslipidemia with <365 days	43/821 (5.24)	109/3284 (3.32)	1.69 (1.17–2.44)	0.005 *	1.64 (1.19–2.25)	0.002 *
Dyslipidemia with ≥365 days	21/821 (2.56)	83/3284 (2.53)	1.08 (0.66–1.77)	0.751	1.14 (0.76–1.70)	0.526

Abbreviations: CCI, Charlson Comorbidity Index; * Significance at *p* < 0.05; † Adjusted for age, sex, income, region of residence, diabetes history, CCI scores, and diabetes.

## Data Availability

Data are contained within the article and Supplementary Materials.

## Supplementary Materials

The following supporting information can be downloaded at: https://www.mdpi.com/article/10.3390/biomedicines11082247/s1, Table S1: Crude and overlap propensity score weighted odds ratios of dates of any statin prescription for any brain tumor, Table S2: Crude and overlap propensity score weighted odds ratios of dates of Lipophilic statin prescription for any brain tumor, Table S3: Crude and overlap propensity score weighted odds ratios of dates of Hydrophilic statin prescription for any brain tumor, Table S4: Crude and overlap propensity score weighted odds ratios of dates of any statin prescription for malignant brain tumors, Table S5: Crude and overlap propensity score weighted odds ratios of dates of Lipophilic statin prescription for malignant brain tumors, Table S6: Crude and overlap propensity score weighted odds ratios of dates of Hydrophilic statin prescription for malignant brain tumors, Table S7: Crude and overlap propensity score weighted odds ratios of dates of any statin prescription for benign brain tumors, Table S8: Crude and overlap propensity score weighted odds ratios of dates of Lipophilic statin prescription for benign brain tumors, Table S9: Crude and overlap propensity score weighted odds ratios of dates of Hydrophilic statin prescription for benign brain tumors.
