# Supplementary material for: Association between the Use of Statins and Brain Tumors
Source: Biomedicines. 2023 Aug 10;11(8):2247. doi: 10.3390/biomedicines11082247 (PMC10452399; doi:10.3390/biomedicines11082247)
Supplement: Supplementary file 1 [file biomedicines-11-02247-s001.zip › S1 (Any statin for any brain tumor).pdf]

Table S1. Crude and overlap propensity score weighted odd ratios of dates of any statin prescription for any brain tumor.

Characteristics	N of Any brain tumor (exposure/total, %)	N of Control (exposure/total, %)	Odd ratios for any brain tumor (95% confidence interval)			
			Crude	P-value	Overlap weighted model †	P-value
Age < 55 years old (n= 4,485)						
Normal	667/897 (74.36)	2,710/3,588 (75.53)	1		1	
Dyslipidemia without Statin	162/897 (18.06)	695/3,588 (19.37)	0.95 (0.78-1.15)	0.576	0.91 (0.78-1.06)	0.217
Dyslipidemia with < 365 days	43/897 (4.79)	108/3,588 (3.01)	1.62 (1.12-2.33)	0.009*	1.91 (1.36-2.66)	<0.001*
Dyslipidemia with ≥ 365 days	25/897 (2.79)	75/3,588 (2.09)	1.35 (0.85-2.15)	0.197	1.19 (0.81-1.76)	0.376
Age ≥ 55 years old (n= 4,980)						
Normal	493/996 (49.5)	1,747/3,984 (43.85)	1		1	
Dyslipidemia without Statin	187/996 (18.78)	1,165/3,984 (29.24)	0.57 (0.47-0.68)	<0.001*	0.68 (0.59-0.78)	<0.001*
Dyslipidemia with < 365 days	141/996 (14.16)	401/3,984 (10.07)	1.25 (1.00-1.55)	0.046*	1.42 (1.18-1.71)	<0.001*
Dyslipidemia with ≥ 365 days	175/996 (17.57)	671/3,984 (16.84)	0.92 (0.76-1.12)	0.426	1.12 (0.96-1.32)	0.161
Male (n= 4,275)						
Normal	565/855 (66.08)	2,175/3,420 (63.6)	1		1	
Dyslipidemia without Statin	149/855 (17.43)	796/3,420 (23.27)	0.72 (0.59-0.88)	0.001*	0.83 (0.71-0.97)	0.019*
Dyslipidemia with < 365 days	70/855 (8.19)	177/3,420 (5.18)	1.52 (1.14-2.04)	0.005*	1.84 (1.42-2.38)	<0.001*
Dyslipidemia with ≥ 365 days	71/855 (8.3)	272/3,420 (7.95)	1.00 (0.76-1.33)	0.973	1.28 (1.02-1.60)	0.032*
Female (n= 5,190)						
Normal	595/1,038 (57.32)	2,282/4,152 (54.96)	1		1	

Dyslipidemia without Statin	200/1,038 (19.27)	1,064/4,152 (25.63)	0.72 (0.60-0.86)	<0.001*	0.79 (0.69-0.91)	0.001*
Dyslipidemia with < 365 days	114/1,038 (10.98)	332/4,152 (8.00)	1.32 (1.05-1.66)	0.019*	1.44 (1.18-1.76)	<0.001*
Dyslipidemia with ≥ 365 days	129/1,038 (12.43)	474/4,152 (11.42)	1.04 (0.84-1.29)	0.695	1.15 (0.95-1.40)	0.154
Low income groups (n= 4,260)						
Normal	550/852 (64.55)	2,096/3,408 (61.5)	1		1	
Dyslipidemia without Statin	143/852 (16.78)	787/3,408 (23.09)	0.69 (0.57-0.85)	<0.001*	0.83 (0.71-0.98)	0.024*
Dyslipidemia with < 365 days	77/852 (9.04)	209/3,408 (6.13)	1.40 (1.06-1.85)	0.017*	1.73 (1.35-2.23)	<0.001*
Dyslipidemia with ≥ 365 days	82/852 (9.62)	316/3,408 (9.27)	0.99 (0.76-1.28)	0.933	1.09 (0.88-1.36)	0.428
High income groups (n= 5,205)						
Normal	610/1,041 (58.6)	2,361/4,164 (56.7)	1		1	
Dyslipidemia without Statin	206/1,041 (19.79)	1,073/4,164 (25.77)	0.74 (0.62-0.88)	<0.001*	0.79 (0.69-0.91)	<0.001*
Dyslipidemia with < 365 days	107/1,041 (10.28)	300/4,164 (7.2)	1.38 (1.09-1.75)	0.008*	1.51 (1.23-1.85)	<0.001*
Dyslipidemia with ≥ 365 days	118/1,041 (11.34)	430/4,164 (10.33)	1.06 (0.85-1.33)	0.595	1.32 (1.09-1.61)	0.005*
Urban residents (n= 4,245)						
Normal	506/849 (59.6)	1,958/3,396 (57.66)	1		1	
Dyslipidemia without Statin	171/849 (20.14)	857/3,396 (25.24)	0.77 (0.64-0.93)	0.008*	0.88 (0.75-1.03)	0.105
Dyslipidemia with < 365 days	78/849 (9.19)	231/3,396 (6.8)	1.31 (0.99-1.72)	0.056	1.63 (1.28-2.07)	<0.001*
Dyslipidemia with ≥ 365 days	94/849 (11.07)	350/3,396 (10.31)	1.04 (0.81-1.33)	0.761	1.16 (0.94-1.45)	0.172
Rural residents (n= 5,220)						
Normal	654/1,044 (62.64)	2,499/4,176 (59.84)	1		1	

Dyslipidemia without Statin	178/1,044 (17.05)	1,003/4,176 (24.02)	0.68 (0.57-0.81)	<0.001*	0.74 (0.64-0.86)	<0.001*
Dyslipidemia with < 365 days	106/1,044 (10.15)	278/4,176 (6.66)	1.46 (1.15-1.85)	0.002*	1.56 (1.27-1.93)	<0.001*
Dyslipidemia with ≥ 365 days	106/1,044 (10.15)	396/4,176 (9.48)	1.02 (0.81-1.29)	0.848	1.28 (1.05-1.56)	0.015*
CCI scores = 0 (n= 6,141)						
Normal	325/558 (58.24)	3,495/5,583 (62.6)	1		1	
Dyslipidemia without Statin	127/558 (22.76)	1,294/5,583 (23.18)	1.06 (0.85-1.31)	0.622	1.04 (0.91-1.19)	0.552
Dyslipidemia with < 365 days	54/558 (9.68)	342/5,583 (6.13)	1.70 (1.25-2.31)	<0.001*	1.70 (1.38-2.10)	<0.001*
Dyslipidemia with ≥ 365 days	52/558 (9.32)	452/5,583 (8.1)	1.24 (0.91-1.68)	0.177	1.30 (1.05-1.60)	0.015*
CCI scores = 1 (n= 1,147)						
Normal	63/149 (42.28)	487/998 (48.8)	1		1	
Dyslipidemia without Statin	33/149 (22.15)	280/998 (28.06)	0.91 (0.58-1.42)	0.682	0.94 (0.70-1.27)	0.699
Dyslipidemia with < 365 days	27/149 (18.12)	89/998 (8.92)	2.35 (1.42-3.88)	<0.001*	2.89 (1.94-4.31)	<0.001*
Dyslipidemia with ≥ 365 days	26/149 (17.45)	142/998 (14.23)	1.42 (0.86-2.32)	0.168	1.98 (1.34-2.92)	<0.001*
CCI scores ≥ 2 (n= 2,177)						
Normal	772/1,186 (65.09)	475/991 (47.93)	1		1	
Dyslipidemia without Statin	189/1,186 (15.94)	286/991 (28.86)	0.41 (0.33-0.50)	<0.001*	0.50 (0.40-0.63)	<0.001*
Dyslipidemia with < 365 days	103/1,186 (8.68)	78/991 (7.87)	0.81 (0.59-1.11)	0.197	1.20 (0.87-1.67)	0.272
Dyslipidemia with ≥ 365 days	122/1,186 (10.29)	152/991 (15.34)	0.49 (0.38-0.64)	<0.001*	0.99 (0.75-1.31)	0.938
Non-diabetes history (n= 7,041)						
Normal	971/1,367 (71.03)	3,897/5,674 (68.68)	1		1	

Dyslipidemia without Statin	215/1,367 (15.73)	1,187/5,674 (20.92)	0.73 (0.62-0.85)	<0.001*	0.83 (0.73-0.94)	0.003*
Dyslipidemia with < 365 days	94/1,367 (6.88)	281/5,674 (4.95)	1.34 (1.05-1.71)	0.018*	1.49 (1.21-1.84)	<0.001*
Dyslipidemia with ≥ 365 days	87/1,367 (6.36)	309/5,674 (5.45)	1.13 (0.88-1.45)	0.334	1.25 (1.02-1.53)	0.034*
Diabetes history (n= 2,424)						
Normal	189/526 (35.93)	560/1,898 (29.5)	1		1	
Dyslipidemia without Statin	134/526 (25.48)	673/1,898 (35.46)	0.59 (0.46-0.76)	<0.001*	0.72 (0.59-0.88)	0.002*
Dyslipidemia with < 365 days	90/526 (17.11)	228/1,898 (12.01)	1.17 (0.87-1.57)	0.297	1.60 (1.23-2.07)	<0.001*
Dyslipidemia with ≥ 365 days	113/526 (21.48)	437/1,898 (23.02)	0.77 (0.59-1.00)	0.048*	1.09 (0.87-1.37)	0.469

Abbreviations: CCI, Charlson Comorbidity Index;

* Significance at $P < 0.05$

† Adjusted for age, sex, income, region of residence, CCI scores and diabetes history.
