# Supplementary material for: Association between the Use of Statins and Brain Tumors
Source: Biomedicines. 2023 Aug 10;11(8):2247. doi: 10.3390/biomedicines11082247 (PMC10452399; doi:10.3390/biomedicines11082247)
Supplement: Supplementary file 1 [file biomedicines-11-02247-s001.zip › S2 (Lipophilic statin for any brain tumor).pdf]

Table S2. Crude and overlap propensity score weighted odd ratios of dates of Lipophilic statin prescription for any brain tumor.

Characteristics	N of Any brain tumor (exposure/total, %)	N of Control (exposure/total, %)	Odd ratios for any brain tumor (95% confidence interval)			
			Crude	P-value	Overlap weighted model †	P-value
Age < 55 years old (n= 4,485)						
Normal	667/897 (74.36)	2,710/3,588 (75.53)	1		1	
Dyslipidemia without Lipophilic statin	176/897 (19.62)	732/3,588 (20.4)	0.98 (0.81-1.18)	0.804	0.94 (0.81-1.09)	0.419
Dyslipidemia with < 365 days	34/897 (3.79)	98/3,588 (2.73)	1.41 (0.95-2.10)	0.092	1.67 (1.16-2.39)	0.005*
Dyslipidemia with ≥ 365 days	20/897 (2.23)	48/3,588 (1.34)	1.69 (1.00-2.87)	0.05	1.37 (0.88-2.14)	0.164
Age ≥ 55 years old (n= 4,980)						
Normal	493/996 (49.5)	1,747/3,984 (43.85)	1		1	
Dyslipidemia without Lipophilic statin	242/996 (24.3)	1,399/3,984 (35.12)	0.61 (0.52-0.73)	<0.001*	0.74 (0.65-0.85)	<0.001*
Dyslipidemia with < 365 days	129/996 (12.95)	363/3,984 (9.11)	1.26 (1.01-1.58)	0.044*	1.40 (1.16-1.70)	<0.001*
Dyslipidemia with ≥ 365 days	132/996 (13.25)	475/3,984 (11.92)	0.98 (0.79-1.22)	0.89	1.17 (0.98-1.40)	0.088
Male (n= 4,275)						
Normal	565/855 (66.08)	2,175/3,420 (63.6)	1		1	
Dyslipidemia without Lipophilic statin	175/855 (20.47)	905/3,420 (26.46)	0.74 (0.62-0.90)	0.002*	0.86 (0.74-1.00)	0.058
Dyslipidemia with < 365 days	63/855 (7.37)	163/3,420 (4.77)	1.49 (1.10-2.02)	0.011*	1.86 (1.42-2.44)	<0.001*
Dyslipidemia with ≥ 365 days	52/855 (6.08)	177/3,420 (5.18)	1.13 (0.82-1.56)	0.455	1.39 (1.07-1.80)	0.014*

Female (n= 5,190)						
Normal	595/1,038 (57.32)	2,282/4,152 (54.96)	1		1	
Dyslipidemia without Lipophilic statin	243/1,038 (23.41)	1,226/4,152 (29.53)	0.76 (0.64-0.90)	0.001*	0.85 (0.74-0.97)	0.017*
Dyslipidemia with < 365 days	100/1,038 (9.63)	298/4,152 (7.18)	1.29 (1.01-1.64)	0.042*	1.35 (1.09-1.66)	0.005*
Dyslipidemia with ≥ 365 days	100/1,038 (9.63)	346/4,152 (8.33)	1.11 (0.87-1.41)	0.401	1.17 (0.95-1.45)	0.139
Low income groups (n= 4,260)						
Normal	550/852 (64.55)	2,096/3,408 (61.5)	1		1	
Dyslipidemia without Lipophilic statin	168/852 (19.72)	912/3,408 (26.76)	0.70 (0.58-0.85)	<0.001*	0.85 (0.73-0.98)	0.031*
Dyslipidemia with < 365 days	70/852 (8.22)	186/3,408 (5.46)	1.43 (1.07-1.92)	0.015*	1.75 (1.35-2.27)	<0.001*
Dyslipidemia with ≥ 365 days	64/852 (7.51)	214/3,408 (6.28)	1.14 (0.85-1.53)	0.384	1.22 (0.96-1.56)	0.11
High income groups (n= 5,205)						
Normal	610/1,041 (58.6)	2,361/4,164 (56.7)	1		1	
Dyslipidemia without Lipophilic statin	250/1,041 (24.02)	1,219/4,164 (29.27)	0.79 (0.67-0.93)	0.005*	0.86 (0.75-0.98)	0.026*
Dyslipidemia with < 365 days	93/1,041 (8.93)	275/4,164 (6.6)	1.31 (1.02-1.68)	0.036*	1.40 (1.13-1.73)	0.002*
Dyslipidemia with ≥ 365 days	88/1,041 (8.45)	309/4,164 (7.42)	1.10 (0.86-1.42)	0.451	1.32 (1.05-1.64)	0.015*
Urban residents (n= 4,245)						
Normal	506/849 (59.6)	1,958/3,396 (57.66)	1		1	
Dyslipidemia without Lipophilic statin	202/849 (23.79)	976/3,396 (28.74)	0.80 (0.67-0.96)	0.016*	0.92 (0.79-1.07)	0.264

Dyslipidemia with < 365 days	70/849 (8.24)	212/3,396 (6.24)	1.28 (0.96-1.70)	0.094	1.59 (1.24-2.03)	<0.001*
Dyslipidemia with ≥ 365 days	71/849 (8.36)	250/3,396 (7.36)	1.10 (0.83-1.46)	0.511	1.19 (0.93-1.52)	0.167
Rural residents (n= 5,220)						
Normal	654/1,044 (62.64)	2,499/4,176 (59.84)	1		1	
Dyslipidemia without Lipophilic statin	216/1,044 (20.69)	1,155/4,176 (27.66)	0.71 (0.60-0.85)	<0.001*	0.80 (0.70-0.92)	0.001*
Dyslipidemia with < 365 days	93/1,044 (8.91)	249/4,176 (5.96)	1.43 (1.11-1.84)	0.006*	1.48 (1.19-1.84)	<0.001*
Dyslipidemia with ≥ 365 days	81/1,044 (7.76)	273/4,176 (6.54)	1.13 (0.87-1.47)	0.349	1.36 (1.09-1.70)	0.007*
CCI scores = 0 (n= 6,141)						
Normal	325/558 (58.24)	3,495/5,583 (62.6)	1		1	
Dyslipidemia without Lipophilic statin	149/558 (26.7)	1,474/5,583 (26.4)	1.09 (0.89-1.33)	0.421	1.08 (0.95-1.23)	0.248
Dyslipidemia with < 365 days	49/558 (8.78)	295/5,583 (5.28)	1.79 (1.29-2.47)	<0.001*	1.78 (1.43-2.23)	<0.001*
Dyslipidemia with ≥ 365 days	35/558 (6.27)	319/5,583 (5.71)	1.18 (0.82-1.70)	0.377	1.22 (0.96-1.56)	0.105
CCI scores = 1 (n= 1,147)						
Normal	63/149 (42.28)	487/998 (48.8)	1		1	
Dyslipidemia without Lipophilic statin	42/149 (28.19)	327/998 (32.77)	0.99 (0.66-1.50)	0.973	1.06 (0.80-1.41)	0.683
Dyslipidemia with < 365 days	22/149 (14.77)	95/998 (9.52)	1.79 (1.05-3.05)	0.032*	2.30 (1.53-3.47)	<0.001*
Dyslipidemia with ≥ 365 days	22/149 (14.77)	89/998 (8.92)	1.91 (1.12-3.26)	0.018*	2.69 (1.74-4.17)	<0.001*
CCI scores ≥ 2 (n= 2,177)						
Normal	772/1,186 (65.09)	475/991 (47.93)	1		1	

Dyslipidemia without Lipophilic statin	227/1,186 (19.14)	330/991 (33.3)	0.42 (0.35-0.52)	<0.001*	0.56 (0.45-0.69)	<0.001*
Dyslipidemia with < 365 days	92/1,186 (7.76)	71/991 (7.16)	0.80 (0.57-1.11)	0.178	1.13 (0.80-1.59)	0.484
Dyslipidemia with ≥ 365 days	95/1,186 (8.01)	115/991 (11.6)	0.51 (0.38-0.68)	<0.001*	1.00 (0.74-1.37)	0.987
Non-diabetes history (n= 7,041)						
Normal	971/1,367 (71.03)	3,897/5,674 (68.68)	1		1	
Dyslipidemia without Lipophilic statin	252/1,367 (18.43)	1,321/5,674 (23.28)	0.77 (0.66-0.89)	<0.001*	0.87 (0.77-0.98)	0.024*
Dyslipidemia with < 365 days	83/1,367 (6.07)	242/5,674 (4.27)	1.38 (1.06-1.78)	0.015*	1.44 (1.16-1.80)	0.001*
Dyslipidemia with ≥ 365 days	61/1,367 (4.46)	214/5,674 (3.77)	1.14 (0.85-1.53)	0.368	1.31 (1.04-1.67)	0.024*
Diabetes history (n= 2,424)						
Normal	189/526 (35.93)	560/1,898 (29.5)	1		1	
Dyslipidemia without Lipophilic statin	166/526 (31.56)	810/1,898 (42.68)	0.61 (0.48-0.77)	<0.001*	0.78 (0.64-0.95)	0.013*
Dyslipidemia with < 365 days	80/526 (15.21)	219/1,898 (11.54)	1.08 (0.80-1.47)	0.61	1.49 (1.14-1.94)	0.003*
Dyslipidemia with ≥ 365 days	91/526 (17.3)	309/1,898 (16.28)	0.87 (0.66-1.16)	0.35	1.15 (0.90-1.47)	0.266

Abbreviations: CCI, Charlson Comorbidity Index;

* Significance at P < 0.05

† Adjusted for age, sex, income, region of residence, CCI scores and diabetes history
