# Supplementary material for: Association between the Use of Statins and Brain Tumors
Source: Biomedicines. 2023 Aug 10;11(8):2247. doi: 10.3390/biomedicines11082247 (PMC10452399; doi:10.3390/biomedicines11082247)
Supplement: Supplementary file 1 [file biomedicines-11-02247-s001.zip › S3 (Hydrophilic statin for any brain tumor).pdf]

Table S3. Crude and overlap propensity score weighted odd ratios of dates of Hydrophilic statin prescription for any brain tumor.

Characteristics	N of Any brain tumor (exposure/total, %)	N of Control (exposure/total, %)	Odd ratios for any brain tumor (95% confidence interval)			
			Crude	P-value	Overlap weighted model †	P-value
Age < 55 years old (n= 4,485)						
Normal	667/897 (74.36)	2,710/3,588 (75.53)	1		1	
Dyslipidemia without Hydrophilic statin	205/897 (22.85)	826/3,588 (23.02)	1.01 (0.85-1.20)	0.926	0.96 (0.83-1.11)	0.613
Dyslipidemia with < 365 days	22/897 (2.45)	28/3,588 (0.78)	3.19 (1.81-5.62)	<0.001*	3.49 (1.99-6.13)	<0.001*
Dyslipidemia with ≥ 365 days	3/897 (0.33)	24/3,588 (0.67)	0.51 (0.15-1.69)	0.27	0.47 (0.18-1.23)	0.123
Age ≥ 55 years old (n= 4,980)						
Normal	493/996 (49.5)	1,747/3,984 (43.85)	1		1	
Dyslipidemia without Hydrophilic statin	402/996 (40.36)	1,872/3,984 (46.99)	0.76 (0.66-0.88)	<0.001*	0.89 (0.79-1.01)	0.075
Dyslipidemia with < 365 days	63/996 (6.33)	194/3,984 (4.87)	1.15 (0.85-1.56)	0.361	1.31 (1.02-1.68)	0.033*
Dyslipidemia with ≥ 365 days	38/996 (3.82)	171/3,984 (4.29)	0.79 (0.55-1.13)	0.2	1.00 (0.75-1.34)	0.984
Male (n= 4,275)						
Normal	565/855 (66.08)	2,175/3,420 (63.6)	1		1	
Dyslipidemia without Hydrophilic statin	240/855 (28.07)	1,085/3,420 (31.73)	0.85 (0.72-1.01)	0.06	0.99 (0.86-1.14)	0.85
Dyslipidemia with < 365 days	35/855 (4.09)	76/3,420 (2.22)	1.77 (1.18-2.67)	0.006*	2.19 (1.52-3.18)	<0.001*
Dyslipidemia with ≥ 365 days	15/855 (1.75)	84/3,420 (2.46)	0.69 (0.39-1.20)	0.187	0.89 (0.59-1.34)	0.588

Female (n= 5,190)						
Normal	595/1,038 (57.32)	2,282/4,152 (54.96)	1		1	
Dyslipidemia without Hydrophilic statin	367/1,038 (35.36)	1,613/4,152 (38.85)	0.87 (0.75-1.01)	0.065	0.94 (0.83-1.06)	0.296
Dyslipidemia with < 365 days	50/1,038 (4.82)	146/4,152 (3.52)	1.31 (0.94-1.83)	0.109	1.34 (1.01-1.78)	0.042*
Dyslipidemia with ≥ 365 days	26/1,038 (2.5)	111/4,152 (2.67)	0.90 (0.58-1.39)	0.63	1.09 (0.75-1.57)	0.658
Low income groups (n= 4,260)						
Normal	550/852 (64.55)	2,096/3,408 (61.5)	1		1	
Dyslipidemia without Hydrophilic statin	250/852 (29.34)	1,128/3,408 (33.1)	0.84 (0.72-1.00)	0.046*	0.98 (0.85-1.13)	0.806
Dyslipidemia with < 365 days	40/852 (4.69)	93/3,408 (2.73)	1.64 (1.12-2.40)	0.011*	1.81 (1.29-2.55)	<0.001*
Dyslipidemia with ≥ 365 days	12/852 (1.41)	91/3,408 (2.67)	0.50 (0.27-0.92)	0.027*	0.60 (0.38-0.93)	0.021*
High income groups (n= 5,205)						
Normal	610/1,041 (58.6)	2,361/4,164 (56.7)	1		1	
Dyslipidemia without Hydrophilic statin	357/1,041 (34.29)	1,570/4,164 (37.7)	0.88 (0.76-1.02)	0.085	0.94 (0.83-1.07)	0.355
Dyslipidemia with < 365 days	45/1,041 (4.32)	129/4,164 (3.1)	1.35 (0.95-1.92)	0.093	1.50 (1.12-2.02)	0.007*
Dyslipidemia with ≥ 365 days	29/1,041 (2.79)	104/4,164 (2.5)	1.08 (0.71-1.64)	0.723	1.40 (0.98-2.00)	0.066
Urban residents (n= 4,245)						
Normal	506/849 (59.6)	1,958/3,396 (57.66)	1		1	
Dyslipidemia without Hydrophilic statin	289/849 (34.04)	1,243/3,396 (36.6)	0.90 (0.77-1.06)	0.198	1.01 (0.88-1.16)	0.863

Dyslipidemia with < 365 days	35/849 (4.12)	106/3,396 (3.12)	1.28 (0.86-1.90)	0.223	1.48 (1.07-2.06)	0.019*
Dyslipidemia with ≥ 365 days	19/849 (2.24)	89/3,396 (2.62)	0.83 (0.50-1.37)	0.458	0.98 (0.66-1.46)	0.924
Rural residents (n= 5,220)						
Normal	654/1,044 (62.64)	2,499/4,176 (59.84)	1		1	
Dyslipidemia without Hydrophilic statin	318/1,044 (30.46)	1,455/4,176 (34.84)	0.84 (0.72-0.97)	0.018*	0.91 (0.81-1.04)	0.163
Dyslipidemia with < 365 days	50/1,044 (4.79)	116/4,176 (2.78)	1.65 (1.17-2.32)	0.004*	1.79 (1.32-2.42)	<0.001*
Dyslipidemia with ≥ 365 days	22/1,044 (2.11)	106/4,176 (2.54)	0.79 (0.50-1.27)	0.331	1.04 (0.72-1.50)	0.846
CCI scores = 0 (n= 6,141)						
Normal	325/558 (58.24)	3,495/5,583 (62.6)	1		1	
Dyslipidemia without Hydrophilic statin	198/558 (35.48)	1,824/5,583 (32.67)	1.17 (0.97-1.41)	0.102	1.15 (1.02-1.30)	0.027*
Dyslipidemia with < 365 days	20/558 (3.58)	146/5,583 (2.62)	1.47 (0.91-2.38)	0.114	1.53 (1.12-2.11)	0.008*
Dyslipidemia with ≥ 365 days	15/558 (2.69)	118/5,583 (2.11)	1.37 (0.79-2.37)	0.264	1.41 (0.99-2.03)	0.06
CCI scores = 1 (n= 1,147)						
Normal	63/149 (42.28)	487/998 (48.8)	1		1	
Dyslipidemia without Hydrophilic statin	66/149 (44.3)	439/998 (43.99)	1.16 (0.80-1.68)	0.424	1.28 (0.98-1.66)	0.072
Dyslipidemia with < 365 days	17/149 (11.41)	29/998 (2.91)	4.53 (2.36-8.71)	<0.001*	5.70 (3.17-10.3)	<0.001*
Dyslipidemia with ≥ 365 days	3/149 (2.01)	43/998 (4.31)	0.54 (0.16-1.79)	0.313	0.66 (0.31-1.41)	0.284
CCI scores ≥ 2 (n= 2,177)						
Normal	772/1,186 (65.09)	475/991 (47.93)	1		1	

Dyslipidemia without Hydrophilic statin	343/1,186 (28.92)	435/991 (43.9)	0.49 (0.40-0.58)	<0.001*	0.68 (0.56-0.82)	<0.001*
Dyslipidemia with < 365 days	48/1,186 (4.05)	47/991 (4.74)	0.63 (0.41-0.95)	0.029*	1.06 (0.68-1.64)	0.803
Dyslipidemia with ≥ 365 days	23/1,186 (1.94)	34/991 (3.43)	0.42 (0.24-0.72)	0.002*	0.86 (0.49-1.50)	0.589
Non-diabetes history (n= 7,041)						
Normal	971/1,367 (71.03)	3,897/5,674 (68.68)	1		1	
Dyslipidemia without Hydrophilic statin	333/1,367 (24.36)	1,575/5,674 (27.76)	0.85 (0.74-0.97)	0.019*	0.96 (0.86-1.07)	0.457
Dyslipidemia with < 365 days	41/1,367 (3)	118/5,674 (2.08)	1.40 (0.97-2.00)	0.072	1.45 (1.07-1.96)	0.015*
Dyslipidemia with ≥ 365 days	22/1,367 (1.61)	84/5,674 (1.48)	1.05 (0.65-1.69)	0.837	1.02 (0.70-1.49)	0.904
Diabetes history (n= 2,424)						
Normal	189/526 (35.93)	560/1,898 (29.5)	1		1	
Dyslipidemia without Hydrophilic statin	274/526 (52.09)	1,123/1,898 (59.17)	0.72 (0.59-0.89)	0.003*	0.92 (0.77-1.10)	0.341
Dyslipidemia with < 365 days	44/526 (8.37)	104/1,898 (5.48)	1.25 (0.85-1.85)	0.255	1.76 (1.24-2.49)	0.001*
Dyslipidemia with ≥ 365 days	19/526 (3.61)	111/1,898 (5.85)	0.51 (0.30-0.85)	0.01*	0.88 (0.59-1.33)	0.549

Abbreviations: CCI, Charlson Comorbidity Index;

* Significance at P < 0.05

† Adjusted for age, sex, income, region of residence, CCI scores and diabetes history
