# Supplementary material for: Association between the Use of Statins and Brain Tumors
Source: Biomedicines. 2023 Aug 10;11(8):2247. doi: 10.3390/biomedicines11082247 (PMC10452399; doi:10.3390/biomedicines11082247)
Supplement: Supplementary file 1 [file biomedicines-11-02247-s001.zip › S4 (Any statin for malignant brain tumor).pdf]

Table S4. Crude and overlap propensity score weighted odd ratios of dates of any statin prescription for malignant brain tumor.

Characteristics	N of Malignant brain tumor (exposure/total, %)	N of Control (exposure/total, %)	Odd ratios for malignant brain tumor (95% confidence interval)			
			Crude	P-value	Overlap weighted model †	P-value
Age < 55 years old (n= 2,505)						
Normal	392/501 (78.24)	1,574/2,004 (78.54)	1		1	
Dyslipidemia without Statin	77/501 (15.37)	344/2,004 (17.17)	0.90 (0.69-1.18)	0.44	0.96 (0.77-1.19)	0.683
Dyslipidemia with < 365 days	19/501 (3.79)	50/2,004 (2.5)	1.53 (0.89-2.62)	0.124	3.15 (1.85-5.38)	<0.001*
Dyslipidemia with ≥ 365 days	13/501 (2.59)	36/2,004 (1.8)	1.45 (0.76-2.76)	0.257	1.11 (0.64-1.94)	0.702
Age ≥ 55 years old (n= 2,855)						
Normal	333/571 (58.32)	1,022/2,284 (44.75)	1		1	
Dyslipidemia without Statin	81/571 (14.19)	651/2,284 (28.5)	0.38 (0.29-0.50)	<0.001*	0.44 (0.36-0.54)	<0.001*
Dyslipidemia with < 365 days	69/571 (12.08)	228/2,284 (9.98)	0.93 (0.69-1.25)	0.625	1.20 (0.94-1.54)	0.146
Dyslipidemia with ≥ 365 days	88/571 (15.41)	383/2,284 (16.77)	0.71 (0.54-0.92)	0.009*	0.92 (0.75-1.14)	0.463
Male (n= 2,745)						
Normal	391/549 (71.22)	1,406/2,196 (64.03)	1		1	
Dyslipidemia without Statin	82/549 (14.94)	507/2,196 (23.09)	0.58 (0.45-0.75)	<0.001*	0.68 (0.56-0.83)	<0.001*
Dyslipidemia with < 365 days	41/549 (7.47)	105/2,196 (4.78)	1.40 (0.96-2.05)	0.078	1.72 (1.25-2.39)	0.001*
Dyslipidemia with ≥ 365 days	35/549 (6.38)	178/2,196 (8.11)	0.71 (0.48-1.03)	0.073	0.93 (0.71-1.23)	0.627
Female (n= 2,615)						
Normal	334/523 (63.86)	1,190/2,092 (56.88)	1		1	

Dyslipidemia without Statin	76/523 (14.53)	488/2,092 (23.33)	0.55 (0.42-0.73)	<0.001*	0.61 (0.49-0.76)	<0.001*
Dyslipidemia with < 365 days	47/523 (8.99)	173/2,092 (8.27)	0.97 (0.69-1.37)	0.853	1.47 (1.09-1.97)	0.012*
Dyslipidemia with ≥ 365 days	66/523 (12.62)	241/2,092 (11.52)	0.98 (0.72-1.31)	0.872	1.23 (0.94-1.60)	0.128
Low income groups (n= 2,520)						
Normal	349/504 (69.25)	1,254/2,016 (62.2)	1		1	
Dyslipidemia without Statin	65/504 (12.9)	448/2,016 (22.22)	0.52 (0.39-0.69)	<0.001*	0.72 (0.58-0.89)	0.003*
Dyslipidemia with < 365 days	44/504 (8.73)	119/2,016 (5.9)	1.33 (0.92-1.91)	0.128	2.01 (1.44-2.81)	<0.001*
Dyslipidemia with ≥ 365 days	46/504 (9.13)	195/2,016 (9.67)	0.85 (0.60-1.19)	0.344	1.07 (0.82-1.40)	0.633
High income groups (n= 2,840)						
Normal	376/568 (66.2)	1,342/2,272 (59.07)	1		1	
Dyslipidemia without Statin	93/568 (16.37)	547/2,272 (24.08)	0.61 (0.47-0.78)	<0.001*	0.59 (0.48-0.72)	<0.001*
Dyslipidemia with < 365 days	44/568 (7.75)	159/2,272 (7.00)	0.99 (0.69-1.41)	0.945	1.33 (0.99-1.78)	0.056
Dyslipidemia with ≥ 365 days	55/568 (9.68)	224/2,272 (9.86)	0.88 (0.64-1.20)	0.414	1.15 (0.88-1.51)	0.315
Urban residents (n= 2,295)						
Normal	309/459 (67.32)	1,101/1,836 (59.97)	1		1	
Dyslipidemia without Statin	69/459 (15.03)	436/1,836 (23.75)	0.56 (0.42-0.75)	<0.001*	0.68 (0.55-0.85)	<0.001*
Dyslipidemia with < 365 days	38/459 (8.28)	107/1,836 (5.83)	1.27 (0.86-1.87)	0.238	2.51 (1.75-3.59)	<0.001*
Dyslipidemia with ≥ 365 days	43/459 (9.37)	192/1,836 (10.46)	0.80 (0.56-1.14)	0.211	0.87 (0.65-1.16)	0.342
Rural residents (n= 3,065)						
Normal	416/613 (67.86)	1,495/2,452 (60.97)	1		1	

Dyslipidemia without Statin	89/613 (14.52)	559/2,452 (22.8)	0.57 (0.45-0.73)	<0.001*	0.61 (0.51-0.74)	<0.001*
Dyslipidemia with < 365 days	50/613 (8.16)	171/2,452 (6.97)	1.05 (0.75-1.47)	0.771	1.19 (0.90-1.58)	0.228
Dyslipidemia with ≥ 365 days	58/613 (9.46)	227/2,452 (9.26)	0.92 (0.67-1.25)	0.587	1.34 (1.04-1.73)	0.024*
CCI scores = 0 (n= 3,238)						
Normal	56/98 (57.14)	2,025/3,140 (64.49)	1		1	
Dyslipidemia without Statin	19/98 (19.39)	691/3,140 (22.01)	0.99 (0.59-1.68)	0.983	1.36 (1.10-1.67)	0.004*
Dyslipidemia with < 365 days	16/98 (16.33)	183/3,140 (5.83)	3.16 (1.78-5.62)	<0.001*	4.68 (3.47-6.32)	<0.001*
Dyslipidemia with ≥ 365 days	7/98 (7.14)	241/3,140 (7.68)	1.05 (0.47-2.33)	0.904	1.78 (1.25-2.52)	0.001*
CCI scores = 1 (n= 597)						
Normal	13/36 (36.11)	285/561 (50.8)	1		1	
Dyslipidemia without Statin	8/36 (22.22)	139/561 (24.78)	1.26 (0.51-3.12)	0.614	1.73 (1.08-2.77)	0.022*
Dyslipidemia with < 365 days	4/36 (11.11)	52/561 (9.27)	1.69 (0.53-5.37)	0.377	2.68 (1.40-5.15)	0.003*
Dyslipidemia with ≥ 365 days	11/36 (30.56)	85/561 (15.15)	2.84 (1.23-6.56)	0.015*	5.67 (3.24-9.92)	<0.001*
CCI scores ≥ 2 (n= 1,525)						
Normal	656/938 (69.94)	286/587 (48.72)	1		1	
Dyslipidemia without Statin	131/938 (13.97)	165/587 (28.11)	0.35 (0.26-0.45)	<0.001*	0.41 (0.31-0.54)	<0.001*
Dyslipidemia with < 365 days	68/938 (7.25)	43/587 (7.33)	0.69 (0.46-1.04)	0.073	1.13 (0.74-1.73)	0.575
Dyslipidemia with ≥ 365 days	83/938 (8.85)	93/587 (15.84)	0.39 (0.28-0.54)	<0.001*	0.78 (0.56-1.10)	0.152
Non-diabetes history (n= 3,984)						
Normal	599/785 (76.31)	2,258/3,199 (70.58)	1		1	

Dyslipidemia without Statin	95/785 (12.1)	619/3,199 (19.35)	0.58 (0.46-0.73)	<0.001*	0.66 (0.55-0.79)	<0.001*
Dyslipidemia with < 365 days	46/785 (5.86)	146/3,199 (4.56)	1.19 (0.84-1.67)	0.326	1.57 (1.17-2.10)	0.003*
Dyslipidemia with ≥ 365 days	45/785 (5.73)	176/3,199 (5.5)	0.96 (0.69-1.35)	0.832	0.89 (0.68-1.16)	0.38
Diabetes history (n= 1,376)						
Normal	126/287 (43.9)	338/1,089 (31.04)	1		1	
Dyslipidemia without Statin	63/287 (21.95)	376/1,089 (34.53)	0.45 (0.32-0.63)	<0.001*	0.56 (0.42-0.73)	<0.001*
Dyslipidemia with < 365 days	42/287 (14.63)	132/1,089 (12.12)	0.85 (0.57-1.28)	0.441	1.42 (0.99-2.02)	0.055
Dyslipidemia with ≥ 365 days	56/287 (19.51)	243/1,089 (22.31)	0.62 (0.43-0.88)	0.008*	1.13 (0.83-1.53)	0.441

Abbreviations: CCI, Charlson Comorbidity Index;

* Significance at P < 0.05

† Adjusted for age, sex, income, region of residence, CCI scores and diabetes history.
