# Supplementary material for: Association between the Use of Statins and Brain Tumors
Source: Biomedicines. 2023 Aug 10;11(8):2247. doi: 10.3390/biomedicines11082247 (PMC10452399; doi:10.3390/biomedicines11082247)
Supplement: Supplementary file 1 [file biomedicines-11-02247-s001.zip › S5 (Lipophilic statin for malignant brain tumor).pdf]

Table S5. Crude and overlap propensity score weighted odd ratios of dates of Lipophilic statin prescription for malignant brain tumor.

Characteristics	N of Malignant brain tumor (exposure/total, %)	N of Control (exposure/total, %)	Odd ratios for malignant brain tumor (95% confidence interval)			
			Crude	P-value	Overlap weighted model †	P-value
Age < 55 years old (n= 2,505)						
Normal	392/501 (78.24)	1,574/2,004 (78.54)	1		1	
Dyslipidemia without Lipophilic statin	82/501 (16.37)	361/2,004 (18.01)	0.91 (0.70-1.19)	0.494	0.99 (0.79-1.22)	0.895
Dyslipidemia with < 365 days	17/501 (3.39)	46/2,004 (2.3)	1.48 (0.84-2.62)	0.173	2.91 (1.66-5.09)	<0.001*
Dyslipidemia with ≥ 365 days	10/501 (2)	23/2,004 (1.15)	1.75 (0.82-3.70)	0.146	1.12 (0.61-2.07)	0.713
Age ≥ 55 years old (n= 2,855)						
Normal	333/571 (58.32)	1,022/2,284 (44.75)	1		1	
Dyslipidemia without Lipophilic statin	107/571 (18.74)	789/2,284 (34.54)	0.42 (0.33-0.53)	<0.001*	0.50 (0.42-0.60)	<0.001*
Dyslipidemia with < 365 days	66/571 (11.56)	207/2,284 (9.06)	0.98 (0.72-1.33)	0.889	1.29 (1.00-1.68)	0.05
Dyslipidemia with ≥ 365 days	65/571 (11.38)	266/2,284 (11.65)	0.75 (0.56-1.01)	0.058	0.94 (0.74-1.19)	0.605
Male (n= 2,745)						
Normal	391/549 (71.22)	1,406/2,196 (64.03)	1		1	
Dyslipidemia without Lipophilic statin	94/549 (17.12)	576/2,196 (26.23)	0.59 (0.46-0.75)	<0.001*	0.67 (0.55-0.81)	<0.001*
Dyslipidemia with < 365 days	37/549 (6.74)	99/2,196 (4.51)	1.34 (0.91-1.99)	0.141	1.98 (1.40-2.80)	<0.001*
Dyslipidemia with ≥ 365 days	27/549 (4.92)	115/2,196 (5.24)	0.84 (0.55-1.30)	0.444	1.06 (0.77-1.45)	0.731

Female (n= 2,615)						
Normal	334/523 (63.86)	1,190/2,092 (56.88)	1		1	
Dyslipidemia without Lipophilic statin	95/523 (18.16)	574/2,092 (27.44)	0.59 (0.46-0.76)	<0.001*	0.71 (0.58-0.87)	<0.001*
Dyslipidemia with < 365 days	46/523 (8.8)	154/2,092 (7.36)	1.06 (0.75-1.51)	0.728	1.44 (1.06-1.96)	0.02*
Dyslipidemia with ≥ 365 days	48/523 (9.18)	174/2,092 (8.32)	0.98 (0.70-1.38)	0.921	1.07 (0.80-1.44)	0.636
Low income groups (n= 2,520)						
Normal	349/504 (69.25)	1,254/2,016 (62.2)	1		1	
Dyslipidemia without Lipophilic statin	77/504 (15.28)	529/2,016 (26.24)	0.52 (0.40-0.68)	<0.001*	0.72 (0.59-0.88)	0.002*
Dyslipidemia with < 365 days	44/504 (8.73)	104/2,016 (5.16)	1.52 (1.05-2.20)	0.027*	2.52 (1.76-3.60)	<0.001*
Dyslipidemia with ≥ 365 days	34/504 (6.75)	129/2,016 (6.4)	0.95 (0.64-1.41)	0.788	1.10 (0.81-1.49)	0.546
High income groups (n= 2,840)						
Normal	376/568 (66.2)	1,342/2,272 (59.07)	1		1	
Dyslipidemia without Lipophilic statin	112/568 (19.72)	621/2,272 (27.33)	0.64 (0.51-0.81)	<0.001*	0.66 (0.55-0.80)	<0.001*
Dyslipidemia with < 365 days	39/568 (6.87)	149/2,272 (6.56)	0.93 (0.64-1.35)	0.719	1.25 (0.92-1.69)	0.15
Dyslipidemia with ≥ 365 days	41/568 (7.22)	160/2,272 (7.04)	0.91 (0.64-1.31)	0.628	1.10 (0.81-1.48)	0.552
Urban residents (n= 2,295)						
Normal	309/459 (67.32)	1,101/1,836 (59.97)	1		1	
Dyslipidemia without Lipophilic statin	82/459 (17.86)	491/1,836 (26.74)	0.60 (0.46-0.78)	<0.001*	0.74 (0.60-0.92)	0.005*

Dyslipidemia with < 365 days	37/459 (8.06)	104/1,836 (5.66)	1.27 (0.85-1.88)	0.24	2.55 (1.76-3.68)	<0.001*
Dyslipidemia with ≥ 365 days	31/459 (6.75)	140/1,836 (7.63)	0.79 (0.52-1.19)	0.256	0.77 (0.55-1.07)	0.122
Rural residents (n= 3,065)						
Normal	416/613 (67.86)	1,495/2,452 (60.97)	1		1	
Dyslipidemia without Lipophilic statin	107/613 (17.46)	659/2,452 (26.88)	0.58 (0.46-0.74)	<0.001*	0.65 (0.54-0.78)	<0.001*
Dyslipidemia with < 365 days	46/613 (7.5)	149/2,452 (6.08)	1.11 (0.78-1.57)	0.558	1.29 (0.95-1.73)	0.099
Dyslipidemia with ≥ 365 days	44/613 (7.18)	149/2,452 (6.08)	1.06 (0.75-1.51)	0.742	1.44 (1.08-1.92)	0.012*
CCI scores = 0 (n= 3,238)						
Normal	56/98 (57.14)	2,025/3,140 (64.49)	1		1	
Dyslipidemia without Lipophilic statin	22/98 (22.45)	788/3,140 (25.1)	1.01 (0.61-1.66)	0.97	1.40 (1.15-1.72)	<0.001*
Dyslipidemia with < 365 days	16/98 (16.33)	161/3,140 (5.13)	3.59 (2.02-6.41)	<0.001*	5.45 (3.97-7.46)	<0.001*
Dyslipidemia with ≥ 365 days	4/98 (4.08)	166/3,140 (5.29)	0.87 (0.31-2.43)	0.793	1.49 (0.97-2.29)	0.066
CCI scores = 1 (n= 597)						
Normal	13/36 (36.11)	285/561 (50.8)	1		1	
Dyslipidemia without Lipophilic statin	10/36 (27.78)	166/561 (29.59)	1.32 (0.57-3.08)	0.519	1.85 (1.18-2.89)	0.007*
Dyslipidemia with < 365 days	5/36 (13.89)	55/561 (9.8)	1.99 (0.68-5.82)	0.207	3.53 (1.86-6.67)	<0.001*
Dyslipidemia with ≥ 365 days	8/36 (22.22)	55/561 (9.8)	3.19 (1.26-8.06)	0.014*	6.10 (3.28-11.4)	<0.001*
CCI scores ≥ 2 (n= 1,525)						
Normal	656/938 (69.94)	286/587 (48.72)	1		1	

Dyslipidemia without Lipophilic statin	157/938 (16.74)	196/587 (33.39)	0.35 (0.27-0.45)	<0.001*	0.46 (0.35-0.60)	<0.001*
Dyslipidemia with < 365 days	62/938 (6.61)	37/587 (6.3)	0.73 (0.48-1.12)	0.153	1.13 (0.71-1.78)	0.608
Dyslipidemia with ≥ 365 days	63/938 (6.72)	68/587 (11.58)	0.40 (0.28-0.58)	<0.001*	0.77 (0.53-1.12)	0.175
Non-diabetes history (n= 3,984)						
Normal	599/785 (76.31)	2,258/3,199 (70.58)	1		1	
Dyslipidemia without Lipophilic statin	108/785 (13.76)	690/3,199 (21.57)	0.59 (0.47-0.74)	<0.001*	0.65 (0.55-0.77)	<0.001*
Dyslipidemia with < 365 days	46/785 (5.86)	130/3,199 (4.06)	1.33 (0.94-1.89)	0.105	1.78 (1.31-2.42)	<0.001*
Dyslipidemia with ≥ 365 days	32/785 (4.08)	121/3,199 (3.78)	1.00 (0.67-1.49)	0.988	0.98 (0.73-1.32)	0.907
Diabetes history (n= 1,376)						
Normal	126/287 (43.9)	338/1,089 (31.04)	1		1	
Dyslipidemia without Lipophilic statin	81/287 (28.22)	460/1,089 (42.24)	0.47 (0.35-0.65)	<0.001*	0.67 (0.52-0.87)	0.002*
Dyslipidemia with < 365 days	37/287 (12.89)	123/1,089 (11.29)	0.81 (0.53-1.23)	0.318	1.32 (0.91-1.92)	0.138
Dyslipidemia with ≥ 365 days	43/287 (14.98)	168/1,089 (15.43)	0.69 (0.46-1.02)	0.06	1.08 (0.77-1.50)	0.66

Abbreviations: CCI, Charlson Comorbidity Index;

* Significance at P < 0.05

† Adjusted for age, sex, income, region of residence, CCI scores and diabetes history
