# Supplementary material for: Association between the Use of Statins and Brain Tumors
Source: Biomedicines. 2023 Aug 10;11(8):2247. doi: 10.3390/biomedicines11082247 (PMC10452399; doi:10.3390/biomedicines11082247)
Supplement: Supplementary file 1 [file biomedicines-11-02247-s001.zip › S6 (Hydrophilic statin for malignant brain tumor).pdf]

Table S6. Crude and overlap propensity score weighted odd ratios of dates of Hydrophilic statin prescription for malignant brain tumor.

Characteristics	N of Malignant brain tumor (exposure/total, %)	N of Control (exposure/total, %)	Odd ratios for malignant brain tumor (95% confidence interval)			
			Crude	P-value	Overlap weighted model †	P-value
Age < 55 years old (n= 2,505)						
Normal	392/501 (78.24)	1,574/2,004 (78.54)	1		1	
Dyslipidemia without Hydrophilic statin	100/501 (19.96)	407/2,004 (20.31)	0.99 (0.77-1.26)	0.914	1.07 (0.87-1.32)	0.493
Dyslipidemia with < 365 days	7/501 (1.4)	13/2,004 (0.65)	2.16 (0.86-5.46)	0.102	2.20 (0.96-5.04)	0.062
Dyslipidemia with ≥ 365 days	2/501 (0.4)	10/2,004 (0.5)	0.80 (0.18-3.68)	0.778	1.10 (0.28-4.37)	0.892
Age ≥ 55 years old (n= 2,855)						
Normal	333/571 (58.32)	1,022/2,284 (44.75)	1		1	
Dyslipidemia without Hydrophilic statin	185/571 (32.4)	1,060/2,284 (46.41)	0.54 (0.44-0.65)	<0.001*	0.66 (0.56-0.78)	<0.001*
Dyslipidemia with < 365 days	35/571 (6.13)	100/2,284 (4.38)	1.07 (0.72-1.61)	0.729	1.41 (0.99-2.01)	0.056
Dyslipidemia with ≥ 365 days	18/571 (3.15)	102/2,284 (4.47)	0.54 (0.32-0.91)	0.02*	0.74 (0.50-1.10)	0.139
Male (n= 2,745)						
Normal	391/549 (71.22)	1,406/2,196 (64.03)	1		1	
Dyslipidemia without Hydrophilic statin	133/549 (24.23)	689/2,196 (31.38)	0.69 (0.56-0.86)	<0.001*	0.83 (0.70-0.99)	0.039*
Dyslipidemia with < 365 days	19/549 (3.46)	48/2,196 (2.19)	1.42 (0.83-2.45)	0.203	1.67 (1.05-2.67)	0.031*
Dyslipidemia with ≥ 365 days	6/549 (1.09)	53/2,196 (2.41)	0.41 (0.17-0.95)	0.039*	0.54 (0.31-0.95)	0.033*

Female (n= 2,615)						
Normal	334/523 (63.86)	1,190/2,092 (56.88)	1		1	
Dyslipidemia without Hydrophilic statin	152/523 (29.06)	778/2,092 (37.19)	0.70 (0.56-0.86)	<0.001*	0.80 (0.67-0.96)	0.015*
Dyslipidemia with < 365 days	23/523 (4.4)	65/2,092 (3.11)	1.26 (0.77-2.06)	0.354	1.75 (1.13-2.71)	0.013*
Dyslipidemia with ≥ 365 days	14/523 (2.68)	59/2,092 (2.82)	0.85 (0.47-1.53)	0.58	1.51 (0.89-2.56)	0.129
Low income groups (n= 2,520)						
Normal	349/504 (69.25)	1,254/2,016 (62.2)	1		1	
Dyslipidemia without Hydrophilic statin	128/504 (25.4)	651/2,016 (32.29)	0.71 (0.56-0.88)	0.002*	0.94 (0.78-1.13)	0.511
Dyslipidemia with < 365 days	21/504 (4.17)	53/2,016 (2.63)	1.42 (0.85-2.39)	0.182	1.79 (1.12-2.85)	0.015*
Dyslipidemia with ≥ 365 days	6/504 (1.19)	58/2,016 (2.88)	0.37 (0.16-0.87)	0.022*	0.57 (0.33-1.00)	0.049*
High income groups (n= 2,840)						
Normal	376/568 (66.2)	1,342/2,272 (59.07)	1		1	
Dyslipidemia without Hydrophilic statin	157/568 (27.64)	816/2,272 (35.92)	0.69 (0.56-0.84)	<0.001*	0.73 (0.61-0.87)	<0.001*
Dyslipidemia with < 365 days	21/568 (3.7)	60/2,272 (2.64)	1.25 (0.75-2.08)	0.392	1.69 (1.09-2.62)	0.02*
Dyslipidemia with ≥ 365 days	14/568 (2.46)	54/2,272 (2.38)	0.93 (0.51-1.68)	0.8	1.43 (0.84-2.43)	0.184
Urban residents (n= 2,295)						
Normal	309/459 (67.32)	1,101/1,836 (59.97)	1		1	
Dyslipidemia without Hydrophilic statin	126/459 (27.45)	643/1,836 (35.02)	0.70 (0.56-0.88)	0.002*	0.87 (0.72-1.06)	0.158

Dyslipidemia with < 365 days	15/459 (3.27)	48/1,836 (2.61)	1.11 (0.62-2.02)	0.722	1.71 (1.05-2.78)	0.03*
Dyslipidemia with ≥ 365 days	9/459 (1.96)	44/1,836 (2.4)	0.73 (0.35-1.51)	0.394	0.98 (0.56-1.71)	0.935
Rural residents (n= 3,065)						
Normal	416/613 (67.86)	1,495/2,452 (60.97)	1		1	
Dyslipidemia without Hydrophilic statin	159/613 (25.94)	824/2,452 (33.61)	0.69 (0.57-0.85)	<0.001*	0.79 (0.67-0.93)	0.005*
Dyslipidemia with < 365 days	27/613 (4.4)	65/2,452 (2.65)	1.49 (0.94-2.37)	0.089	1.80 (1.17-2.75)	0.007*
Dyslipidemia with ≥ 365 days	11/613 (1.79)	68/2,452 (2.77)	0.58 (0.30-1.11)	0.1	0.87 (0.53-1.44)	0.585
CCI scores = 0 (n= 3,238)						
Normal	56/98 (57.14)	2,025/3,140 (64.49)	1		1	
Dyslipidemia without Hydrophilic statin	36/98 (36.73)	977/3,140 (31.11)	1.33 (0.87-2.04)	0.186	1.82 (1.51-2.18)	<0.001*
Dyslipidemia with < 365 days	4/98 (4.08)	73/3,140 (2.32)	1.98 (0.70-5.61)	0.198	2.93 (1.77-4.84)	<0.001*
Dyslipidemia with ≥ 365 days	2/98 (2.04)	65/3,140 (2.07)	1.11 (0.27-4.66)	0.884	1.66 (0.91-3.03)	0.097
CCI scores = 1 (n= 597)						
Normal	13/36 (36.11)	285/561 (50.8)	1		1	
Dyslipidemia without Hydrophilic statin	18/36 (50)	234/561 (41.71)	1.69 (0.81-3.51)	0.163	2.41 (1.59-3.63)	<0.001*
Dyslipidemia with < 365 days	3/36 (8.33)	18/561 (3.21)	3.65 (0.95-14.0)	0.059	6.81 (2.81-16.5)	<0.001*
Dyslipidemia with ≥ 365 days	2/36 (5.56)	24/561 (4.28)	1.83 (0.39-8.57)	0.445	2.81 (1.17-6.75)	0.021*
CCI scores ≥ 2 (n= 1,525)						
Normal	656/938 (69.94)	286/587 (48.72)	1		1	

Dyslipidemia without Hydrophilic statin	231/938 (24.63)	256/587 (43.61)	0.39 (0.31-0.49)	<0.001*	0.54 (0.43-0.69)	<0.001*
Dyslipidemia with < 365 days	35/938 (3.73)	22/587 (3.75)	0.69 (0.40-1.20)	0.193	1.23 (0.68-2.21)	0.49
Dyslipidemia with ≥ 365 days	16/938 (1.71)	23/587 (3.92)	0.30 (0.16-0.58)	<0.001*	0.72 (0.36-1.45)	0.36
Non-diabetes history (n= 3,984)						
Normal	599/785 (76.31)	2,258/3,199 (70.58)	1		1	
Dyslipidemia without Hydrophilic statin	157/785 (20)	840/3,199 (26.26)	0.70 (0.58-0.85)	<0.001*	0.81 (0.70-0.94)	0.007*
Dyslipidemia with < 365 days	19/785 (2.42)	52/3,199 (1.63)	1.38 (0.81-2.35)	0.238	1.24 (0.80-1.93)	0.344
Dyslipidemia with ≥ 365 days	10/785 (1.27)	49/3,199 (1.53)	0.77 (0.39-1.53)	0.454	0.58 (0.34-0.98)	0.042*
Diabetes history (n= 1,376)						
Normal	126/287 (43.9)	338/1,089 (31.04)	1		1	
Dyslipidemia without Hydrophilic statin	128/287 (44.6)	627/1,089 (57.58)	0.55 (0.41-0.72)	<0.001*	0.76 (0.60-0.96)	0.023*
Dyslipidemia with < 365 days	23/287 (8.01)	61/1,089 (5.6)	1.01 (0.60-1.70)	0.966	2.00 (1.22-3.29)	0.006*
Dyslipidemia with ≥ 365 days	10/287 (3.48)	63/1,089 (5.79)	0.43 (0.21-0.86)	0.016*	1.09 (0.61-1.94)	0.765

Abbreviations: CCI, Charlson Comorbidity Index;

* Significance at P < 0.05

† Adjusted for age, sex, income, region of residence, CCI scores and diabetes history
