# Supplementary material for: Association between the Use of Statins and Brain Tumors
Source: Biomedicines. 2023 Aug 10;11(8):2247. doi: 10.3390/biomedicines11082247 (PMC10452399; doi:10.3390/biomedicines11082247)
Supplement: Supplementary file 1 [file biomedicines-11-02247-s001.zip › S7 (Any statin for benign brain tumor).pdf]

S7 table Crude and overlap propensity score weighted odd ratios of dates of any statin prescription for benign brain tumor

Characteristics	N of Benign brain tumor	N of Control	Odd ratios for benign brain tumor (95% confidence interval)			
	(exposure/total, %)	(exposure/total, %)	Crude	P-value	Overlap weighted model †	P-value
Age < 55 years old (n= 1,980)						
Normal	275/396 (69.44)	1,136/1,584 (71.72)	1		1	
Dyslipidemia without Statin	85/396 (21.46)	351/1,584 (22.16)	1.00 (0.76-1.31)	0.998	0.94 (0.75-1.17)	0.564
Dyslipidemia with < 365 days	24/396 (6.06)	58/1,584 (3.66)	1.71 (1.04-2.80)	0.033*	1.64 (1.05-2.55)	0.03*
Dyslipidemia with ≥ 365 days	12/396 (3.03)	39/1,584 (2.46)	1.27 (0.66-2.46)	0.476	1.19 (0.69-2.07)	0.535
Age ≥ 55 years old (n= 2,125)						
Normal	160/425 (37.65)	725/1,700 (42.65)	1		1	
Dyslipidemia without Statin	106/425 (24.94)	514/1,700 (30.24)	0.93 (0.71-1.22)	0.623	0.95 (0.77-1.18)	0.658
Dyslipidemia with < 365 days	72/425 (16.94)	173/1,700 (10.18)	1.89 (1.36-2.61)	<0.001*	1.80 (1.37-2.38)	<0.001*
Dyslipidemia with ≥ 365 days	87/425 (20.47)	288/1,700 (16.94)	1.37 (1.02-1.84)	0.037*	1.37 (1.07-1.76)	0.012*
Male (n= 1,530)						
Normal	174/306 (56.86)	769/1,224 (62.83)	1		1	
Dyslipidemia without Statin	67/306 (21.9)	289/1,224 (23.61)	1.02 (0.75-1.40)	0.879	1.12 (0.86-1.46)	0.386
Dyslipidemia with < 365 days	29/306 (9.48)	72/1,224 (5.88)	1.78 (1.12-2.82)	0.014*	2.08 (1.37-3.15)	<0.001*
Dyslipidemia with ≥ 365 days	36/306 (11.76)	94/1,224 (7.68)	1.69 (1.11-2.57)	0.014*	1.97 (1.35-2.89)	<0.001*
Female (n= 2,575)						
Normal	261/515 (50.68)	1,092/2,060 (53.01)	1		1	

Dyslipidemia without Statin	124/515 (24.08)	576/2,060 (27.96)	0.90 (0.71-1.14)	0.386	0.89 (0.73-1.08)	0.233
Dyslipidemia with < 365 days	67/515 (13.01)	159/2,060 (7.72)	1.76 (1.29-2.42)	<0.001*	1.62 (1.22-2.14)	<0.001*
Dyslipidemia with ≥ 365 days	63/515 (12.23)	233/2,060 (11.31)	1.13 (0.83-1.54)	0.435	1.10 (0.84-1.45)	0.486
Low income groups (n= 1,740)						
Normal	201/348 (57.76)	842/1,392 (60.49)	1		1	
Dyslipidemia without Statin	78/348 (22.41)	339/1,392 (24.35)	0.96 (0.72-1.29)	0.804	0.94 (0.74-1.20)	0.631
Dyslipidemia with < 365 days	33/348 (9.48)	90/1,392 (6.47)	1.54 (1.00-2.36)	0.049*	1.56 (1.07-2.28)	0.022*
Dyslipidemia with ≥ 365 days	36/348 (10.34)	121/1,392 (8.69)	1.25 (0.83-1.86)	0.284	1.19 (0.83-1.70)	0.338
High income groups (n= 2,365)						
Normal	234/473 (49.47)	1,019/1,892 (53.86)	1		1	
Dyslipidemia without Statin	113/473 (23.89)	526/1,892 (27.8)	0.94 (0.73-1.20)	0.598	0.96 (0.78-1.17)	0.677
Dyslipidemia with < 365 days	63/473 (13.32)	141/1,892 (7.45)	1.95 (1.40-2.70)	<0.001*	1.88 (1.40-2.52)	<0.001*
Dyslipidemia with ≥ 365 days	63/473 (13.32)	206/1,892 (10.89)	1.33 (0.97-1.83)	0.075	1.45 (1.09-1.92)	0.011*
Urban residents (n= 1,950)						
Normal	197/390 (50.51)	857/1,560 (54.94)	1		1	
Dyslipidemia without Statin	102/390 (26.15)	421/1,560 (26.99)	1.05 (0.81-1.38)	0.699	1.06 (0.85-1.32)	0.604
Dyslipidemia with < 365 days	40/390 (10.26)	124/1,560 (7.95)	1.40 (0.95-2.07)	0.087	1.40 (1.00-1.96)	0.047*
Dyslipidemia with ≥ 365 days	51/390 (13.08)	158/1,560 (10.13)	1.40 (0.99-2.00)	0.058	1.45 (1.06-2.00)	0.022*
Rural residents (n= 2,155)						
Normal	238/431 (55.22)	1,004/1,724 (58.24)	1		1	

Dyslipidemia without Statin	89/431 (20.65)	444/1,724 (25.75)	0.85 (0.65-1.11)	0.22	0.86 (0.69-1.07)	0.185
Dyslipidemia with < 365 days	56/431 (12.99)	107/1,724 (6.21)	2.21 (1.55-3.14)	<0.001*	2.15 (1.55-2.97)	<0.001*
Dyslipidemia with ≥ 365 days	48/431 (11.14)	169/1,724 (9.8)	1.20 (0.84-1.70)	0.312	1.25 (0.92-1.70)	0.158
CCI scores = 0 (n= 2,903)						
Normal	269/460 (58.48)	1,470/2,443 (60.17)	1		1	
Dyslipidemia without Statin	108/460 (23.48)	603/2,443 (24.68)	0.98 (0.77-1.25)	0.862	0.98 (0.81-1.19)	0.849
Dyslipidemia with < 365 days	38/460 (8.26)	159/2,443 (6.51)	1.31 (0.90-1.90)	0.165	1.33 (0.99-1.80)	0.062
Dyslipidemia with ≥ 365 days	45/460 (9.78)	211/2,443 (8.64)	1.17 (0.82-1.65)	0.387	1.19 (0.89-1.58)	0.238
CCI scores = 1 (n= 550)						
Normal	50/113 (44.25)	202/437 (46.22)	1		1	
Dyslipidemia without Statin	25/113 (22.12)	141/437 (32.27)	0.72 (0.42-1.21)	0.214	0.72 (0.47-1.09)	0.122
Dyslipidemia with < 365 days	23/113 (20.35)	37/437 (8.47)	2.51 (1.37-4.60)	0.003*	3.09 (1.74-5.50)	<0.001*
Dyslipidemia with ≥ 365 days	15/113 (13.27)	57/437 (13.04)	1.06 (0.56-2.03)	0.853	1.46 (0.81-2.61)	0.204
CCI scores ≥ 2 (n= 652)						
Normal	116/248 (46.77)	189/404 (46.78)	1		1	
Dyslipidemia without Statin	58/248 (23.39)	121/404 (29.95)	0.78 (0.53-1.15)	0.213	0.90 (0.61-1.32)	0.587
Dyslipidemia with < 365 days	35/248 (14.11)	35/404 (8.66)	1.63 (0.97-2.75)	0.067	1.87 (1.09-3.19)	0.022*
Dyslipidemia with ≥ 365 days	39/248 (15.73)	59/404 (14.6)	1.08 (0.68-1.72)	0.755	1.56 (0.96-2.56)	0.074
Non-diabetes history (n= 3,057)						
Normal	372/582 (63.92)	1,639/2,475 (66.22)	1		1	

Dyslipidemia without Statin	120/582 (20.62)	568/2,475 (22.95)	0.93 (0.74-1.17)	0.536	0.99 (0.82-1.18)	0.88
Dyslipidemia with < 365 days	48/582 (8.25)	135/2,475 (5.45)	1.57 (1.11-2.22)	0.011*	1.63 (1.21-2.20)	0.001*
Dyslipidemia with ≥ 365 days	42/582 (7.22)	133/2,475 (5.37)	1.39 (0.97-2.00)	0.076	1.60 (1.17-2.19)	0.003*
Diabetes history (n= 1,048)						
Normal	63/239 (26.36)	222/809 (27.44)	1		1	
Dyslipidemia without Statin	71/239 (29.71)	297/809 (36.71)	0.84 (0.58-1.23)	0.378	0.93 (0.68-1.28)	0.647
Dyslipidemia with < 365 days	48/239 (20.08)	96/809 (11.87)	1.76 (1.13-2.75)	0.013*	1.91 (1.29-2.82)	0.001*
Dyslipidemia with ≥ 365 days	57/239 (23.85)	194/809 (23.98)	1.04 (0.69-1.56)	0.867	1.14 (0.80-1.61)	0.473

Abbreviations: CCI, Charlson Comorbidity Index;

* Significance at $P < 0.05$

† Adjusted for age, sex, income, region of residence, CCI scores and diabetes history.
