# Supplementary material for: Association between the Use of Statins and Brain Tumors
Source: Biomedicines. 2023 Aug 10;11(8):2247. doi: 10.3390/biomedicines11082247 (PMC10452399; doi:10.3390/biomedicines11082247)
Supplement: Supplementary file 1 [file biomedicines-11-02247-s001.zip › S8 (Lipophilic statin for benign brain tumor).pdf]

S8 table Crude and overlap propensity score weighted odd ratios of dates of Lipophilic statin prescription for benign brain tumor

Characteristics	N of Benign brain tumor (exposure/total, %)	N of Control (exposure/total, %)	Odd ratios for benign brain tumor (95% confidence interval)			
			Crude	P-value	Overlap weighted model †	P-value
Age < 55 years old (n= 1,980)						
Normal	275/396 (69.44)	1,136/1,584 (71.72)	1		1	
Dyslipidemia without Lipophilic statin	94/396 (23.74)	371/1,584 (23.42)	1.05 (0.81-1.36)	0.733	0.97 (0.78-1.21)	0.805
Dyslipidemia with < 365 days	17/396 (4.29)	52/1,584 (3.28)	1.35 (0.77-2.37)	0.296	1.36 (0.83-2.23)	0.22
Dyslipidemia with ≥ 365 days	10/396 (2.53)	25/1,584 (1.58)	1.65 (0.79-3.48)	0.186	1.49 (0.79-2.81)	0.221
Age ≥ 55 years old (n= 2,125)						
Normal	160/425 (37.65)	725/1,700 (42.65)	1		1	
Dyslipidemia without Lipophilic statin	135/425 (31.76)	610/1,700 (35.88)	1.00 (0.78-1.29)	0.983	1.03 (0.84-1.27)	0.751
Dyslipidemia with < 365 days	63/425 (14.82)	156/1,700 (9.18)	1.83 (1.30-2.57)	<0.001*	1.69 (1.27-2.26)	<0.001*
Dyslipidemia with ≥ 365 days	67/425 (15.76)	209/1,700 (12.29)	1.45 (1.05-2.01)	0.024*	1.44 (1.09-1.90)	0.009*
Male (n= 1,530)						
Normal	174/306 (56.86)	769/1,224 (62.83)	1		1	
Dyslipidemia without Lipophilic statin	81/306 (26.47)	329/1,224 (26.88)	1.09 (0.81-1.46)	0.573	1.21 (0.94-1.55)	0.139
Dyslipidemia with < 365 days	26/306 (8.5)	64/1,224 (5.23)	1.80 (1.11-2.91)	0.018*	2.05 (1.32-3.17)	0.001*
Dyslipidemia with ≥ 365 days	25/306 (8.17)	62/1,224 (5.07)	1.78 (1.09-2.92)	0.022*	2.08 (1.33-3.25)	0.001*

Female (n= 2,575)						
Normal	261/515 (50.68)	1,092/2,060 (53.01)	1		1	
Dyslipidemia without Lipophilic statin	148/515 (28.74)	652/2,060 (31.65)	0.95 (0.76-1.19)	0.651	0.94 (0.78-1.13)	0.504
Dyslipidemia with < 365 days	54/515 (10.49)	144/2,060 (6.99)	1.57 (1.12-2.21)	0.009*	1.42 (1.06-1.91)	0.02*
Dyslipidemia with ≥ 365 days	52/515 (10.1)	172/2,060 (8.35)	1.26 (0.90-1.77)	0.173	1.21 (0.90-1.64)	0.21
Low income groups (n= 1,740)						
Normal	201/348 (57.76)	842/1,392 (60.49)	1		1	
Dyslipidemia without Lipophilic statin	91/348 (26.15)	383/1,392 (27.51)	1.00 (0.76-1.31)	0.973	0.98 (0.78-1.24)	0.868
Dyslipidemia with < 365 days	26/348 (7.47)	82/1,392 (5.89)	1.33 (0.83-2.12)	0.234	1.33 (0.89-1.99)	0.163
Dyslipidemia with ≥ 365 days	30/348 (8.62)	85/1,392 (6.11)	1.48 (0.95-2.30)	0.084	1.39 (0.94-2.07)	0.101
High income groups (n= 2,365)						
Normal	234/473 (49.47)	1,019/1,892 (53.86)	1		1	
Dyslipidemia without Lipophilic statin	138/473 (29.18)	598/1,892 (31.61)	1.00 (0.80-1.27)	0.967	1.03 (0.85-1.25)	0.738
Dyslipidemia with < 365 days	54/473 (11.42)	126/1,892 (6.66)	1.87 (1.32-2.65)	<0.001*	1.76 (1.29-2.39)	<0.001*
Dyslipidemia with ≥ 365 days	47/473 (9.94)	149/1,892 (7.88)	1.37 (0.96-1.96)	0.082	1.46 (1.06-2.00)	0.02*
Urban residents (n= 1,950)						
Normal	197/390 (50.51)	857/1,560 (54.94)	1		1	
Dyslipidemia without Lipophilic statin	120/390 (30.77)	485/1,560 (31.09)	1.08 (0.84-1.39)	0.568	1.09 (0.88-1.34)	0.431

Dyslipidemia with < 365 days	33/390 (8.46)	108/1,560 (6.92)	1.33 (0.87-2.02)	0.184	1.34 (0.94-1.91)	0.107
Dyslipidemia with ≥ 365 days	40/390 (10.26)	110/1,560 (7.05)	1.58 (1.07-2.35)	0.022*	1.62 (1.13-2.32)	0.009*
Rural residents (n= 2,155)						
Normal	238/431 (55.22)	1,004/1,724 (58.24)	1		1	
Dyslipidemia without Lipophilic statin	109/431 (25.29)	496/1,724 (28.77)	0.93 (0.72-1.19)	0.554	0.95 (0.77-1.17)	0.645
Dyslipidemia with < 365 days	47/431 (10.9)	100/1,724 (5.8)	1.98 (1.36-2.88)	<0.001*	1.85 (1.32-2.59)	<0.001*
Dyslipidemia with ≥ 365 days	37/431 (8.58)	124/1,724 (7.19)	1.26 (0.85-1.87)	0.252	1.28 (0.91-1.81)	0.155
CCI scores = 0 (n= 2,903)						
Normal	269/460 (58.48)	1,470/2,443 (60.17)	1		1	
Dyslipidemia without Lipophilic statin	127/460 (27.61)	686/2,443 (28.08)	1.01 (0.80-1.27)	0.921	1.02 (0.85-1.22)	0.849
Dyslipidemia with < 365 days	33/460 (7.17)	134/2,443 (5.49)	1.35 (0.90-2.01)	0.148	1.35 (0.98-1.87)	0.066
Dyslipidemia with ≥ 365 days	31/460 (6.74)	153/2,443 (6.26)	1.11 (0.74-1.66)	0.624	1.11 (0.81-1.54)	0.511
CCI scores = 1 (n= 550)						
Normal	50/113 (44.25)	202/437 (46.22)	1		1	
Dyslipidemia without Lipophilic statin	32/113 (28.32)	161/437 (36.84)	0.80 (0.49-1.31)	0.38	0.84 (0.56-1.25)	0.392
Dyslipidemia with < 365 days	17/113 (15.04)	40/437 (9.15)	1.72 (0.90-3.28)	0.101	2.14 (1.19-3.85)	0.011*
Dyslipidemia with ≥ 365 days	14/113 (12.39)	34/437 (7.78)	1.66 (0.83-3.33)	0.151	2.36 (1.22-4.56)	0.01*
CCI scores ≥ 2 (n= 652)						
Normal	116/248 (46.77)	189/404 (46.78)	1		1	

Dyslipidemia without Lipophilic statin	70/248 (28.23)	134/404 (33.17)	0.85 (0.59-1.23)	0.393	1.00 (0.69-1.45)	1
Dyslipidemia with < 365 days	30/248 (12.1)	34/404 (8.42)	1.44 (0.84-2.47)	0.19	1.62 (0.94-2.80)	0.082
Dyslipidemia with ≥ 365 days	32/248 (12.9)	47/404 (11.63)	1.11 (0.67-1.84)	0.687	1.59 (0.93-2.72)	0.089
Non-diabetes history (n= 3,057)						
Normal	372/582 (63.92)	1,639/2,475 (66.22)	1		1	
Dyslipidemia without Lipophilic statin	144/582 (24.74)	631/2,475 (25.49)	1.01 (0.81-1.24)	0.96	1.07 (0.90-1.27)	0.454
Dyslipidemia with < 365 days	37/582 (6.36)	112/2,475 (4.53)	1.46 (0.99-2.15)	0.058	1.47 (1.06-2.03)	0.022*
Dyslipidemia with ≥ 365 days	29/582 (4.98)	93/2,475 (3.76)	1.37 (0.89-2.12)	0.149	1.61 (1.11-2.33)	0.011*
Diabetes history (n= 1,048)						
Normal	63/239 (26.36)	222/809 (27.44)	1		1	
Dyslipidemia without Lipophilic statin	85/239 (35.56)	350/809 (43.26)	0.86 (0.59-1.23)	0.405	0.95 (0.70-1.29)	0.757
Dyslipidemia with < 365 days	43/239 (17.99)	96/809 (11.87)	1.58 (1.00-2.49)	0.049*	1.72 (1.16-2.56)	0.007*
Dyslipidemia with ≥ 365 days	48/239 (20.08)	141/809 (17.43)	1.20 (0.78-1.85)	0.408	1.28 (0.88-1.86)	0.197

Abbreviations: CCI, Charlson Comorbidity Index;

* Significance at $P < 0.05$

† Adjusted for age, sex, income, region of residence, CCI scores and diabetes history
