# Supplementary material for: Association between the Use of Statins and Brain Tumors
Source: Biomedicines. 2023 Aug 10;11(8):2247. doi: 10.3390/biomedicines11082247 (PMC10452399; doi:10.3390/biomedicines11082247)
Supplement: Supplementary file 1 [file biomedicines-11-02247-s001.zip › S9 (Hydrophilic statin for benign brain tumor).pdf]

S9 table Crude and overlap propensity score weighted odd ratios of dates of Hydrophilic statin prescription for benign brain tumor

Characteristics	N of Benign brain tumor	N of Control	Odd ratios for benign brain tumor (95% confidence interval)			
	(exposure/total, %)	(exposure/total, %)	Crude	P-value	Overlap weighted model †	P-value
Age < 55 years old (n= 1,980)						
Normal	275/396 (69.44)	1,136/1,584 (71.72)	1		1	
Dyslipidemia without Hydrophilic statin	105/396 (26.52)	419/1,584 (26.45)	1.04 (0.81-1.33)	0.787	0.97 (0.78-1.20)	0.757
Dyslipidemia with < 365 days	15/396 (3.79)	15/1,584 (0.95)	4.13 (2.00-8.55)	<0.001*	3.93 (1.86-8.32)	<0.001*
Dyslipidemia with ≥ 365 days	1/396 (0.25)	14/1,584 (0.88)	0.30 (0.04-2.25)	0.24	0.28 (0.06-1.19)	0.085
Age ≥ 55 years old (n= 2,125)						
Normal	160/425 (37.65)	725/1,700 (42.65)	1		1	
Dyslipidemia without Hydrophilic statin	217/425 (51.06)	812/1,700 (47.76)	1.21 (0.96-1.52)	0.099	1.20 (1.00-1.45)	0.055
Dyslipidemia with < 365 days	28/425 (6.59)	94/1,700 (5.53)	1.35 (0.86-2.13)	0.197	1.32 (0.91-1.90)	0.146
Dyslipidemia with ≥ 365 days	20/425 (4.71)	69/1,700 (4.06)	1.31 (0.78-2.22)	0.31	1.35 (0.88-2.08)	0.168
Male (n= 1,530)						
Normal	174/306 (56.86)	769/1,224 (62.83)	1		1	
Dyslipidemia without Hydrophilic statin	107/306 (34.97)	396/1,224 (32.35)	1.19 (0.91-1.56)	0.197	1.31 (1.03-1.66)	0.029*
Dyslipidemia with < 365 days	16/306 (5.23)	28/1,224 (2.29)	2.53 (1.34-4.77)	0.004*	2.97 (1.63-5.41)	<0.001*
Dyslipidemia with ≥ 365 days	9/306 (2.94)	31/1,224 (2.53)	1.28 (0.60-2.74)	0.52	1.44 (0.77-2.71)	0.253

Female (n= 2,575)						
Normal	261/515 (50.68)	1,092/2,060 (53.01)	1		1	
Dyslipidemia without Hydrophilic statin	215/515 (41.75)	835/2,060 (40.53)	1.08 (0.88-1.32)	0.47	1.03 (0.86-1.22)	0.746
Dyslipidemia with < 365 days	27/515 (5.24)	81/2,060 (3.93)	1.39 (0.88-2.20)	0.153	1.27 (0.86-1.86)	0.228
Dyslipidemia with ≥ 365 days	12/515 (2.33)	52/2,060 (2.52)	0.97 (0.51-1.83)	0.915	0.97 (0.58-1.63)	0.915
Low income groups (n= 1,740)						
Normal	201/348 (57.76)	842/1,392 (60.49)	1		1	
Dyslipidemia without Hydrophilic statin	122/348 (35.06)	477/1,392 (34.27)	1.07 (0.83-1.38)	0.591	1.04 (0.83-1.30)	0.748
Dyslipidemia with < 365 days	19/348 (5.46)	40/1,392 (2.87)	1.99 (1.13-3.51)	0.017*	1.88 (1.13-3.13)	0.015*
Dyslipidemia with ≥ 365 days	6/348 (1.72)	33/1,392 (2.37)	0.76 (0.31-1.84)	0.546	0.74 (0.37-1.47)	0.387
High income groups (n= 2,365)						
Normal	234/473 (49.47)	1,019/1,892 (53.86)	1		1	
Dyslipidemia without Hydrophilic statin	200/473 (42.28)	754/1,892 (39.85)	1.16 (0.94-1.43)	0.18	1.15 (0.96-1.38)	0.121
Dyslipidemia with < 365 days	24/473 (5.07)	69/1,892 (3.65)	1.52 (0.93-2.46)	0.094	1.49 (0.98-2.25)	0.059
Dyslipidemia with ≥ 365 days	15/473 (3.17)	50/1,892 (2.64)	1.31 (0.72-2.37)	0.378	1.43 (0.87-2.35)	0.161
Urban residents (n= 1,950)						
Normal	197/390 (50.51)	857/1,560 (54.94)	1		1	
Dyslipidemia without Hydrophilic statin	163/390 (41.79)	600/1,560 (38.46)	1.18 (0.94-1.49)	0.159	1.17 (0.95-1.43)	0.132

Dyslipidemia with < 365 days	20/390 (5.13)	58/1,560 (3.72)	1.50 (0.88-2.55)	0.134	1.45 (0.92-2.27)	0.11
Dyslipidemia with ≥ 365 days	10/390 (2.56)	45/1,560 (2.88)	0.97 (0.48-1.95)	0.925	1.02 (0.58-1.78)	0.956
Rural residents (n= 2,155)						
Normal	238/431 (55.22)	1,004/1,724 (58.24)	1		1	
Dyslipidemia without Hydrophilic statin	159/431 (36.89)	631/1,724 (36.6)	1.06 (0.85-1.33)	0.593	1.06 (0.87-1.28)	0.576
Dyslipidemia with < 365 days	23/431 (5.34)	51/1,724 (2.96)	1.90 (1.14-3.17)	0.014*	1.86 (1.19-2.93)	0.007*
Dyslipidemia with ≥ 365 days	11/431 (2.55)	38/1,724 (2.2)	1.22 (0.62-2.42)	0.568	1.29 (0.73-2.29)	0.376
CCI scores = 0 (n= 2,903)						
Normal	269/460 (58.48)	1,470/2,443 (60.17)	1		1	
Dyslipidemia without Hydrophilic statin	162/460 (35.22)	847/2,443 (34.67)	1.05 (0.85-1.29)	0.683	1.04 (0.88-1.24)	0.637
Dyslipidemia with < 365 days	16/460 (3.48)	73/2,443 (2.99)	1.20 (0.69-2.09)	0.525	1.24 (0.81-1.90)	0.33
Dyslipidemia with ≥ 365 days	13/460 (2.83)	53/2,443 (2.17)	1.34 (0.72-2.49)	0.354	1.37 (0.84-2.25)	0.205
CCI scores = 1 (n= 550)						
Normal	50/113 (44.25)	202/437 (46.22)	1		1	
Dyslipidemia without Hydrophilic statin	48/113 (42.48)	205/437 (46.91)	0.95 (0.61-1.47)	0.805	1.02 (0.70-1.48)	0.933
Dyslipidemia with < 365 days	14/113 (12.39)	11/437 (2.52)	5.14 (2.20-12.0)	<0.001*	6.20 (2.54-15.1)	<0.001*
Dyslipidemia with ≥ 365 days	1/113 (0.88)	19/437 (4.35)	0.21 (0.03-1.63)	0.136	0.27 (0.07-1.09)	0.066
CCI scores ≥ 2 (n= 652)						
Normal	116/248 (46.77)	189/404 (46.78)	1		1	

Dyslipidemia without Hydrophilic statin	112/248 (45.16)	179/404 (44.31)	1.02 (0.73-1.42)	0.909	1.21 (0.86-1.70)	0.272
Dyslipidemia with < 365 days	13/248 (5.24)	25/404 (6.19)	0.85 (0.42-1.72)	0.647	1.08 (0.53-2.18)	0.835
Dyslipidemia with ≥ 365 days	7/248 (2.82)	11/404 (2.72)	1.04 (0.39-2.75)	0.942	1.47 (0.56-3.83)	0.435
Non-diabetes history (n= 3,057)						
Normal	372/582 (63.92)	1,639/2,475 (66.22)	1		1	
Dyslipidemia without Hydrophilic statin	176/582 (30.24)	735/2,475 (29.7)	1.06 (0.86-1.29)	0.598	1.12 (0.95-1.32)	0.173
Dyslipidemia with < 365 days	22/582 (3.78)	66/2,475 (2.67)	1.47 (0.90-2.41)	0.128	1.53 (1.01-2.32)	0.045*
Dyslipidemia with ≥ 365 days	12/582 (2.06)	35/2,475 (1.41)	1.51 (0.78-2.94)	0.224	1.61 (0.92-2.83)	0.097
Diabetes history (n= 1,048)						
Normal	63/239 (26.36)	222/809 (27.44)	1		1	
Dyslipidemia without Hydrophilic statin	146/239 (61.09)	496/809 (61.31)	1.04 (0.74-1.45)	0.831	1.13 (0.85-1.50)	0.393
Dyslipidemia with < 365 days	21/239 (8.79)	43/809 (5.32)	1.72 (0.95-3.11)	0.072	1.83 (1.09-3.10)	0.024*
Dyslipidemia with ≥ 365 days	9/239 (3.77)	48/809 (5.93)	0.66 (0.31-1.42)	0.288	0.80 (0.43-1.48)	0.481

Abbreviations: CCI, Charlson Comorbidity Index;

* Significance at P < 0.05

† Adjusted for age, sex, income, region of residence, CCI scores and diabetes history
